# Identification of a miRNA multi-targeting therapeutic strategy in glioblastoma

**DOI:** 10.1038/s41419-023-06117-z

**Published:** 2023-09-25

**Authors:** Arthur Bassot, Helena Dragic, Sarah Al Haddad, Laurine Moindrot, Soline Odouard, Francesca Corlazzoli, Eliana Marinari, Alexandra Bomane, Augustin Brassens, Antoine Marteyn, Youssef Hibaoui, Tom J. Petty, Mounira Chalabi-Dchar, Louis Larrouquere, Evgeny M. Zdobnov, Noémie Legrand, Jérôme Tamburini, Hubert Lincet, Marie Castets, Mayra Yebra, Denis Migliorini, Valérie Dutoit, Paul R. Walker, Olivier Preynat-Seauve, Pierre-Yves Dietrich, Érika Cosset

**Affiliations:** 1 Department of CITI, Team GLIMMER Of lIght, Cancer Research Centre of Lyon – CRCL, INSERM U1052, CNRS UMR 5286, Lyon, France; 2 Laboratory of Tumor Immunology, Department of Oncology, Geneva University Hospitals, Geneva, Switzerland; 3 Center for Translational Research in Onco-Hematology, Department of Oncology, University Hospital of Geneva, University of Geneva, Geneva, Switzerland; 4 Swiss Cancer Center Leman (SCCL), Agora Cancer Research Center, Geneva and Lausanne, Switzerland; 5 Department of CITI, Team Cell Death and Chilhood Cancers, Cancer Research Centre of Lyon – CRCL, INSERM U1052, CNRS UMR 5286, Lyon, France; 6 Service de Gynécologie Obstétrique, HFR Fribourg - Hôpital Cantonal, Fribourg, Switzerland; 7 Swiss Institute of Bioinformatics, Geneva, Switzerland; 8 Department of CITI, Team Ribosome, Translation & Cancer, Cancer Research Centre of Lyon – CRCL, INSERM U1052, CNRS UMR 5286, Lyon, France; 9 Department of Genetic Medicine and Development, University of Geneva, and Swiss Institute of Bioinformatics, Geneva, Switzerland; 10 Department of Surgery, Moores Cancer Center, University of California San Diego, La Jolla, CA 92037 USA; 11 Laboratory of Immunobiology of Brain Tumors, Center for Translational Research in OncoHematology, Geneva University Hospitals, and University of Geneva, Geneva, Switzerland; 12 Department of Pathology and Immunology, Medical School, University of Geneva, Geneva, Switzerland; 13 Present Address: SOPHiA GENETICS, Rolle, Switzerland; 14 Present Address: Team: GLIMMER Of lIght “GLIoblastoma MetabolisM, HetERogeneity, and OrganoIds”; Cancer Research Centre of Lyon – CRCL, INSERM U1052, CNRS UMR 5286, Lyon, France

**Keywords:** CNS cancer, RNAi

## Abstract

Glioblastoma (GBM) is a deadly and the most common primary brain tumor in adults. Due to their regulation of a high number of mRNA transcripts, microRNAs (miRNAs) are key molecules in the control of biological processes and are thereby promising therapeutic targets for GBM patients. In this regard, we recently reported miRNAs as strong modulators of GBM aggressiveness. Here, using an integrative and comprehensive analysis of the TCGA database and the transcriptome of GBM biopsies, we identified three critical and clinically relevant miRNAs for GBM, miR-17-3p, miR-222, and miR-340. In addition, we showed that the combinatorial modulation of three of these miRNAs efficiently inhibited several biological processes in patient-derived GBM cells of all these three GBM subtypes (Mesenchymal, Proneural, Classical), induced cell death, and delayed tumor growth in a mouse tumor model. Finally, in a doxycycline-inducible model, we observed a significant inhibition of GBM stem cell viability and a significant delay of orthotopic tumor growth. Collectively, our results reveal, for the first time, the potential of miR-17-3p, miR-222 and miR-340 multi-targeting as a promising therapeutic strategy for GBM patients.

## Introduction

Glioblastoma (GBM) is a grade IV astrocytic glioma, a deadly malignant brain tumor and among the most common primary brain tumors in adults [1]. Today, surgery, radiotherapy, and chemotherapy with temozolomide remain the standard of care for patients with GBM [2]. However, the median overall survival of patients with GBM (~16 months) has not radically changed over the past 15 years. Major efforts in large-scale genomic and transcriptomic profiling have allowed for the characterization and stratification of GBM patients into four subtypes: Proneural, Classical, Mesenchymal, and Neural (which has been now removed) [3–5]. Nevertheless, these big data analyses have yet to highlight new therapeutic avenues and druggable molecules to achieve advances in precision medicine.

MiRNAs are small non-coding RNAs consisting of 20–22 nucleotides that participate in the post-translational regulation of gene expression through RNA interference processes [6–8]. The miRNA genes are transcribed by RNA polymerase II to form pri-miRNA transcripts. These pri-miRNA are processed by Drosha (a class 2 RNAse III enzyme) to release the pre-miRNA precursor product consisting of approximately 70 nucleotides. Finally, the pre-miRNA is exported to the cytoplasm where it is processed to generate a mature ~20-nucleotide miRNA. This miRNA is integrated into the RISC complex (an endoribonuclease of the RNase III family) and forms double-stranded RNA when binding the complementary target mRNAs. As a consequence, a cleavage at the loop‐end of the miRNA structure generates the 5p and 3p strands, where 5p and 3p define whether the miRNA originates from the 5′ or 3′ end of a pre-miRNA hairpin, respectively. Based on genomic and functional experiments, miRNA activity in humans is mainly attributed to 5p strands. Indeed, 3p strands are much less abundant in RISC and are rapidly removed. Depending on miRNAs’ complementation with the target mRNAs, the RISC complex inhibits mRNA translation or induces mRNA degradation [8, 9].

Consistent with recent computational predictions, each miRNA has the potential to regulate about 200 target genes; thus miRNA-mediated gene regulation is now considered to have an important role in biological processes [10]. MiRNAs undergo aberrant expression during tumorigenesis and miRNA encoding genes are frequently located at fragile sites, in regions gained and lost in mammalian cancers [11]. To date, several studies have addressed the role of miRNAs in GBM biology, prognosis or classification. Profiling studies have shown that miRNAs are differentially expressed in brain tumors compared to normal tissues and such dysregulated miRNA subsets have the potential to be used for diagnostic purposes [11]. Several dysregulated miRNAs have been characterized with regard to their function and targets in gliomagenesis. In GBM, a total of 17 upregulated and 33 downregulated miRNAs have been identified from three independent expression profiling studies [12–14]. Notably, miR-10b, miR-21, miR-124, miR-128-1, miR-137, miR-139, miR-218, and miR-323 were found to be dysregulated [15]. Among these, expression profiling revealed that miR-21 is one of the most frequently upregulated miRNAs in GBM. Moreover, functional studies showed that miR-21 knockdown in GBM cells led to decreased cell growth, enhanced apoptosis, reduced invasiveness, and suppressed tumorigenicity [16]. These findings are obtained through microarray expression analysis and allow us to better understand GBM biology. However, more investigation is clearly needed to identify new drivers of GBM aggressiveness and clinically and biologically relevant miRNAs for GBM therapeutic strategies.

Because they play critical roles in various vitally important cellular processes, the use of miRNAs in personalized cancer therapy is extremely attractive. As previously described, some overexpressed miRNAs have been reported to exert “oncogenic” effects, while some downregulated miRNA showed “tumor suppressor” effects. Specific miRNA alterations can be specifically targeted by using oligonucleotide sequences referred to as “mimics” or “antagomirs” that induce an upregulation or downregulation of the targeted miRNAs, respectively. Therefore, by identifying the altered miRNAs in the tumor, targeted therapy for can be used personalized medicine in GBM patients.

In this study, by harnessing the wealth of public data stored in the Cancer Genome Atlas Research Network (TCGA) database, we identified miR-17-3p, miR-222, and miR-340 as three clinically relevant miRNAs that can be used for a GBM multitargeting therapeutic strategy. GBM cell aggressiveness is differentially modulated by the individual miRNAs. Indeed, when used separately, we cannot achieve an inhibition of GBM aggressiveness in all patient-derived models. However, the combination of all three miRNAs shows a systematic and significant inhibition of tumor growth in all subtypes of GBM, in vitro and in vivo, suggesting that multitargeting therapy using miR-222, miR-17-3p, and miR-340 is a promising approach for GBM treatment.

## Results

### Expression of miR-17-3p, miR-222, miR-340, and miR-551b correlates with GBM patient survival

**Fig. 1:**
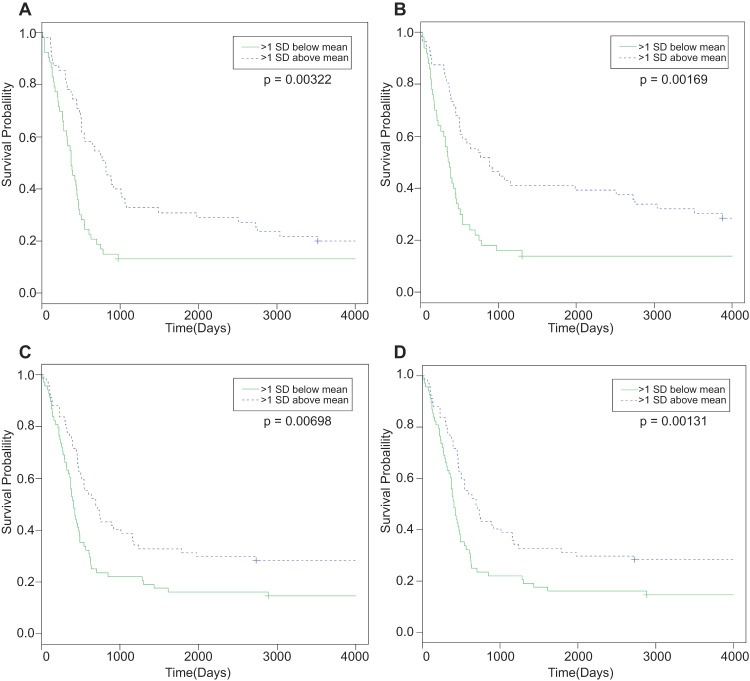
TCGA analysis reveals miR-17-3p, miR-222, miR-340 and miR551b as the four miRNAs for which expression is consistently correlated with poor survival regardless of disease stage, patient age, gender, mutations, treatment. **A** Kaplan–Meier analysis of TCGA dataset for miR-17-3p expression (median = 8.364, mean = 7.969; SD = 1.047, *n* = 53 miR-17-3plow, *n* = 55 miR-17-3phigh; Chi2 = 8.7 on 1 degree of freedom). **B** Kaplan–Meier analysis of TCGA dataset for miR-340 expression (median = 6.992, mean = 6.717; SD = 0.686, *n* = 50 miR-340low, *n* = 56 miR340high; Chi2 = 10.4 on 1 degree of freedom). **C** Kaplan–Meier analysis of TCGA dataset for miR-551b expression (median = 6.720, mean = 7.310; SD = 1.038, *n* = 68 miR-551blow, *n* = 67 miR-551bhigh; Chi2 = 7.3 on 1 degree of freedom). **D** Kaplan–Meier analysis of TCGA dataset for miR-222 expression (median = 8.325, mean = 9.446; SD = 2.066, *n* = 70 miR-222low, *n* = 65 miR-222high; Chi2 = 10.3 on 1 degree of freedom).

To identify clinically relevant miRNAs, we considered GBM plasticity as a strong component of its biology. Therefore, we reasoned that miRNAs for which expression is correlated with patient survival before and after treatments are critical miRNAs for GBM biology. Indeed, a strong phenotypic plasticity of GBM cells has been described upon recurrence: TMZ treatment (as well as radiotherapy) can induce hypermethylation and immunological microenvironment changes [17]. Moreover, post-treatment tumors can switch subtypes (from a classical or proneural toward a more mesenchymal subtype) [17]. Consequently, we grouped the patients from the Cancer Genome Atlas (TCGA) dataset, which represents the largest GBM dataset encompassing all miRNAs, into two subpopulations: (A) patients who received a treatment (any kind of chemotherapy and/or radiotherapy) and (B) patients who did not receive any treatment (Table 1). Then, we selected miRNAs whose expression was significantly correlated with the overall survival of GBM patients. Interestingly, these data outline different points: (i) important miRNAs in the group without treatment are mostly different from those found in the group of GBM patients who received therapy, highlighting the tumor plasticity and complexity; (ii) only four miRNAs (miR-17-3p, miR-222, miR-340 and miR-551b) important for the overall survival of GBM patients without treatment are still critical after treatment (Fig. 1A–D, Supplementary Fig. S1A–D, and Supplementary Table 2). Remarkably, expression of miR-17-3p, miR-340 and miR-551b is higher in patients with longer overall survival, whereas the expression of miR-222 is correlated with worse survival. miR-340 and miR-222 were already identified in previous studies as critical miRNAs whose expression was correlated with better and worse survival in GBM, respectively [12, 18, 19]. In contrast, miR-551b and miR-17-3p expression was not previously associated with GBM survival. Moreover, in the Bao dataset, we could not find any correlation between expression of each miRNA with the others, except for miR-340 and miR-551b, for which expression was significantly and positively correlated (Supplementary Fig. S1E) [20]. Then, by using a bigger dataset, the TCGA dataset, the nearest neighbor analysis showed the relationship between miR-17-3p and miR-340 (Supplementary Fig. S2A). Moreover, this analysis revealed a correlation between miR-17-3p and the other members of its cluster, miR18a, miR-92a, and mir-19b. The latter, together with miR-181a, was also associated with miR340. We also observed a correlation between miR-222 and miR-221, as well as miR-21, all known as oncomiRs in GBM. Finally, we found a greater expression of miR-222 compared to miR-17-3p and miR-340 in GBM patients (Supplementary Fig. S2B).

**Table 1:** TCGA analysis reveals four miRNAs for which expression correlates with GBM patient survival at diagnosis with and without treatment.

All			No treatment			With treatment		
Name	chi2	pval	Name	chi2	pval	Name	chi2	pval
hsa-miR-34a	15	0.00011	hsa-miR-18a*	18.9	1.39E−005	**hsa-miR-17-3p**	14.3	0.000
hsa-miR-218	14.8	0.00012	hsa-miR-551a	13.8	0.000	hsa-miR-200b	11	0.001
hsa-miR-196a	10.5	0.00117	hsa-miR-609	11.9	0.001	hsa-miR-155	10.5	0.001
**hsa-miR-340**	10.4	0.00129	hsa-miR-302c	11.1	0.001	hsa-miR-218	9.3	0.002
**hsa-miR-222**	10.3	0.00131	hsa-miR-483	9.7	0.002	hsa-miR-196a	8.1	0.004
hsa-miR-200b	10.2	0.00142	hsa-miR-767-5p	9	0.003	hsa-miR-361	7.9	0.005
hsa-miR-221	10.2	0.00143	hsa-miR-514	8	0.005	hsa-miR-140	7.8	0.005
**hsa-miR-17-3p**	8.7	0.00322	hsa-miR-615	6.8	0.009	hsa-miR-34a	7.4	0.006
hsa-miR-155	8.1	0.00452	ebv-miR-BART14-5p	6.3	0.012	hsa-miR-92	6.7	0.010
hsa-miR-148a	7.8	0.00532	hsa-miR-206	6	0.014	hsa-miR-199b	6.5	0.011
kshv-miR-K12-2	7.7	0.00551	hsa-miR-647	6	0.014	**hsa-miR-340**	6.4	0.011
**hsa-miR-551b**	7.3	0.00698	hsa-miR-663	5.8	0.016	hsa-miR-148a	6.3	0.012
hsa-miR-101	6.8	0.00925	hsa-miR-571	5.8	0.016	hsa-miR-191	5.4	0.020
hsa-miR-10a	6.7	0.0094	hsa-miR-453	5.7	0.017	**hsa-miR-222**	5.3	0.021
hsa-miR-7	6.3	0.0118	hcmv-miR-UL70-5p	5.6	0.018	hsa-miR-10a	5.3	0.022
hsa-miR-140	6	0.0142	hsa-miR-199a	5.6	0.018	hsa-miR-101	5	0.025
hsa-miR-124a	5.7	0.0173	hsa-miR-21	5.6	0.018	hsa-miR-494	5	0.025
hsa-miR-200a	5.5	0.0187	hsa-miR-105	5.3	0.022	hsa-miR-498	4.9	0.027
hsa-miR-196b	5.3	0.0219	hsa-miR-657	5.1	0.024	hsa-miR-363	4.5	0.034
hsa-miR-34c	5.2	0.0222	hsa-miR-515-3p	5	0.025	hsa-miR-409-3p	4.5	0.034
hsa-miR-30e-3p	4.6	0.0326	hsa-miR-524*	5	0.026	hsa-miR-335	4.4	0.036
hsa-miR-92	4.5	0.0333	hsa-miR-583	4.9	0.027	hsa-miR-197	4.3	0.038
hsa-miR-487b	4.5	0.0345	**hsa-miR-222**	4.9	0.027	**hsa-miR-551b**	4.3	0.038
hsa-miR-34b	4.4	0.0356	hsa-miR-623	4.8	0.028	hsa-miR-196b	4.2	0.040
hsa-miR-133a	4.4	0.0357	ebv-miR-BART2	4.8	0.028	hsa-miR-30e-3p	4.2	0.041
hsa-miR-199b	4.2	0.0414	**hsa-miR-551b**	4.8	0.028	hsa-miR-522	4.1	0.043
hcmv-miR-UL70-5p	4.1	0.0425	hsa-miR-539	4.7	0.030	hsa-miR-520e	3.8	0.050
hsa-miR-526c	4	0.0451	hsa-miR-505	4.6	0.031			
hsa-miR-204	4	0.0454	hsa-miR-642	4.6	0.032			
hsa-miR-30e-5p	4	0.0468	hsa-miR-137	4.4	0.035			
hsa-miR-566	3.9	0.0485	kshv-miR-K12-4-5p	4.3	0.038			
			hsa-miR-638	4.2	0.040			
			hsa-miR-565	4.2	0.041			
			hsa-miR-661	4	0.045			
			**hsa-miR-17-3p**	4	0.045			
			kshv-miR-K12-6-5p	4	0.046			
			hsa-miR-767-3p	4	0.046			
			hsa-miR-15b	4	0.047			
			hsa-miR-520d*	3.7	0.05			
			**hsa-miR-340**	3.7	0.05			

Left column, “All” represents the TCGA analysis without considering any criteria (gender, treatment, age, subtypes, mutations…). In the middle, “No treatment” indicates the list of miRNAs found in the condition without treatment. Right column stands for with treatments. miR-18a*, -524*, and -520d* indicate that miRNAs come from the same hairpin miRNA (from the opposite arm of the precursor) of hsa-miR-18a, -524, and -520d, respectively. The common miRNAs are highlighted in bold. Pval <0.05 was considered as significant.

*Chi2* chi-square value, *pval* *p* value.

### The combinatorial modulation of miR-17-3p, miR-222, and miR-340 inhibits GBM cell viability, clonogenicity, and transmigration capacity and induces cell death

We sought to investigate the therapeutic potential of targeting the identified miRNAs by modulating their expression in GBM patient-derived cells (PDCs). Given that GBM patients who overexpress miR-17-3p, miR-340 and miR-551b have shown greater overall survival whereas, in contrast, GBM patients who overexpress miR-222 have poor overall survival, we selected PDCs where miR-17-3p, miR-340, and miR-551b were not expressed or expressed at a low level, and where miR-222 was expressed. Indeed, we aimed at increasing miR-17-3p, miR-340 and miR-551b expression and decreasing miR-222 expression. After screening nine patient-derived GBM cells and two established cell lines (U87MG and U251) using quantitative RT-PCR (RT-qPCR), we determined that Ge518 was the best PDC model for modulating miRNA expression (Supplementary Table 3).

**Fig. 2:**
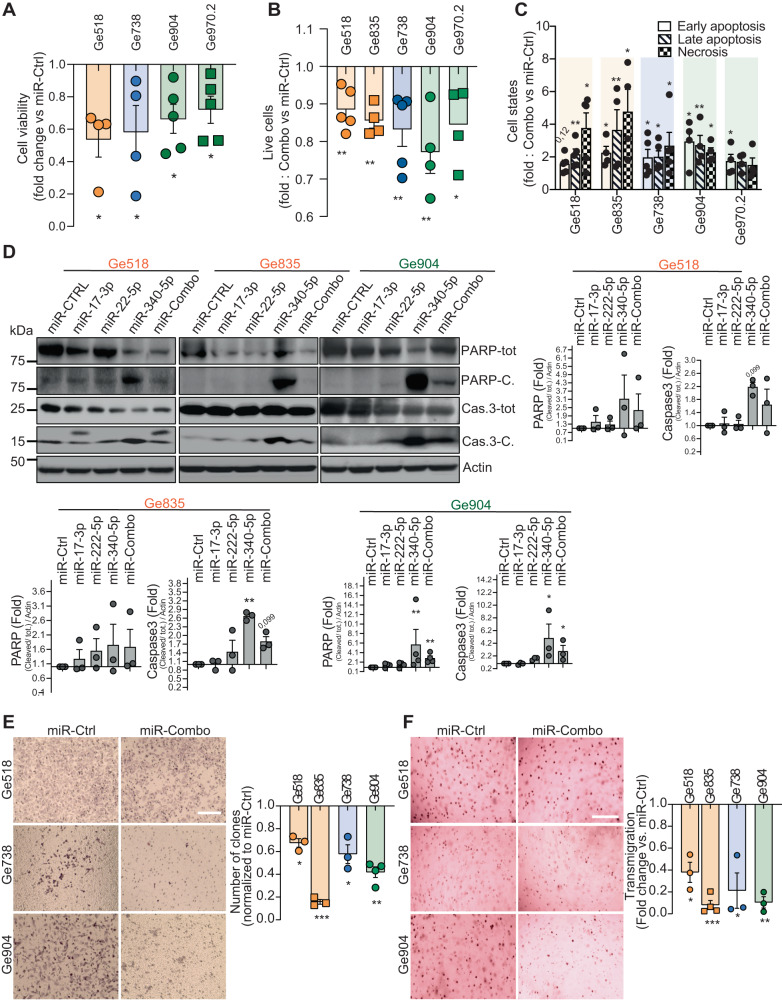
MiR-17-3p, -340, and -222 modulate GBM cell survival, clonogenicity, and transmigration. **A** Cell viability of Ge518, Ge738, Ge904 and Ge970.2 transiently transfected with nontargeting scrambled control or mimics of miR-340, -17-3p, 551b and -222 antagomir was evaluated after three or four days using CellTiter-Glo. Histograms represent the fold change of cell survival for the miR-Combo versus the miR-Ctrl (*n* = 4–5). **B** Histograms represent the fold change of live cells in GDCs transiently transfected with nontargeting scrambled control or miR-Combo evaluated by flow cytometry with Annexin V/PI staining (*n* = 3–4). **C** Histograms represent the fold change of early and late apoptosis and necrosis in GDCs transiently transfected with non-targeting scrambled control or miR-Combo assessed by flow cytometry with Annexin V/PI staining. **D** Protein expression was determined by western blot in Ge518, Ge835 and Ge904 transfected with non-targeting scrambled control or miR-Combo. Histogram represents the fold change of protein expression quantified by using ImageJ (*n* = 4). Data are represented as mean ± SEM (*p < 0.05, **p < 0.01), ns non-significant. **E** Clonogenicity of Ge518, Ge738, Ge904 and Ge970.2 transiently transfected with nontargeting scrambled control or mimics of miR-340, -17-3p, and -222 antagomir was determined using the clonogenic assay. Representative pictures of 3–4 independent experiments. Histograms represent the fold change of clones formed in for miR-Combo versus the miR-Ctrl condition. Scale bar = 10 µm. **F** Transmigration of Ge518, Ge738, Ge904 and Ge970.2 transiently transfected with nontargeting scrambled control or mimics of miR-340, -17-3p, and -222 antagomir was determined using transwell plates. Representative pictures of 3 independent experiments. Histograms represent the fold change of transmigrated cells through the transwell for the miR-Combo versus the miR-Ctrl condition. Scale bar = 10 µm. Data are represented as mean ± SEM (*p < 0.05, **p < 0.01 and ***p < 0.001), ns non-significant.

We reasoned that a miRNA-based multi-targeting therapy would be more beneficial for GBM patients than a single agent therapy. To test this hypothesis, combined mimics and antagomirs (miR-Combo) were used in Ge518 PDCs to either ectopically express or inhibit expression, respectively. After confirming miRNA modulation by quantitative stem-loop real-time RT-PCR (Supplementary Fig. S3A), we measured cell viability at three days post-transfection (Fig. 2A) and observed a significant decrease of cell viability with miR-Combo compared to the non-targeting scrambled control (miR-Ctrl). As previously reported, Ge518 showed a mesenchymal phenotype [21]. To confirm the inhibitory potential of the miR-Combo on other patient-derived models with proneural and classical phenotypes, we also tested cell viability of Ge738 (proneural subtype), Ge904 and Ge970.2 (classical subtype) [21] after transfection (Fig. 2A). Our results revealed a significant inhibition of cell viability for all PDCs, indicating that GBM cells from any subtype can be therapeutically targeted with the miR-Combo. In order to understand which miRNA was responsible for this inhibition, we transfected each miRNA separately, confirmed its modulation (Supplementary Figs. S3G–N and S4A–H), and analyzed PDC viability. As shown in Supplementary Fig. S4I–L, we observed a significant decrease of cell viability of several PDCs mainly through miR-17-3p and miR-340. A trend toward an inhibition of cell viability was shown for miR-222 in Ge970.2 and a significant inhibition was found in Ge518 (Supplementary Fig. S4I–L). Unfortunately, a trend toward an increase of cell viability was found for miR-551b in two PDCs (except for Ge518), indicating that this miRNA might not be a strong candidate and consequently can be removed from the combinatorial strategy (Supplementary Fig. S4K). Furthermore, in order to better understand a rationale behind the cell viability decrease, we performed cell cycle and apoptosis/necrosis analysis. Our data revealed a significant increase of cell apoptosis following miRNA transfection (Supplementary Fig. S4M). Moreover, Annexin V/PI double staining was used to validate this increase of (early and late) apoptotic cells as well as damaged or living cells as shown in Fig. 2B, C. Consistent with this result, we also observed that the miR-Combo induced apoptosis through activation of the caspase-3 pathway, as revealed through a higher expression of both cleaved-Caspase-3 and PARP by immunoblotting (Fig. 2D and Supplementary Fig. S4N) and a slight increase of the G0/G1 cell cycle arrest (Supplementary Fig. S4O).

Then, to better characterize the biological effects of the miR-Combo, we also analyzed the transmigration and clonogenicity potential of GBM PDCs. The miR-Combo induced a significant decrease of cell clonogenicity and transmigration in all PDCs (Fig. 2E, F). Similarly, after the transfection of each miRNA separately, we showed that miR-17-3p, miR-340, and miR-222 mediated most of the observed biological effects and had different effects on different PDCs, highlighting the need to use them all in the combinatorial strategy (Supplementary Fig. S5A–H). Altogether, our data demonstrate that miR-17-3p, miR-340 and miR-222, but not miR-551b, consistently inhibit GBM aggressiveness by affecting their survival, clonogenicity and transmigration capacity.

### Modulation of miR-17-3p, miR-222, and miR-340 regulates genes involved in cell viability

**Fig. 3:**
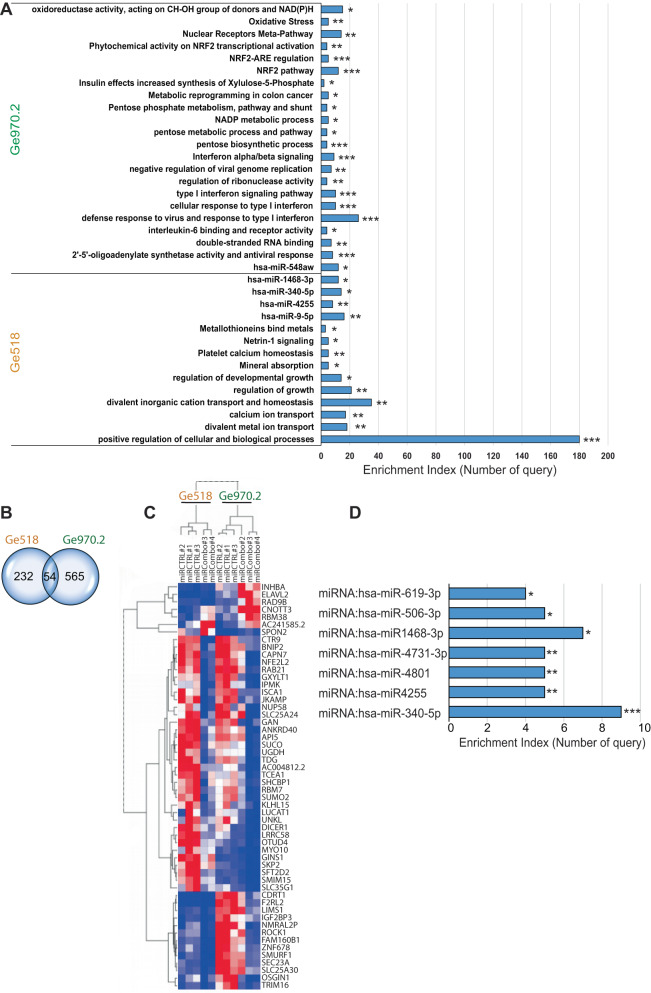
The combinatorial modulation of miR-340, -17-3p, and -222 regulated genes involved in several biological processes and metabolic pathways. **A** Functional annotation clustering of gene set enrichment analysis showing Ge518 and Ge970.2 transfected with a non-targeting scrambled control or the combinatorial modulation of miR-340, -17-3p, and -222. Histograms show the number of query enriched for each family of genes from g:Profiler analysis. **B** Venn diagram of comparisons between Ge518 and Ge970.2 transfected with a nontargeting scrambled control or the combinatorial modulation of miR-340, -17-3p and -222. **C** Hierarchical clustering of Ge518 and Ge970.2 miR-Ctrl vs. miR-Combo based on the differentially expressed genes. **D** Functional annotation clustering of gene set enrichment analysis comparing Ge518 and Ge970.2 transfected with a non-targeting scrambled control or the combinatorial modulation of miR-340, -17-3p and -222. Histograms show the number of queries enriched for each family of genes from g:Profiler analysis. Data are represented as mean ± SEM (*p < 0.05, **p < 0.01 and ***p < 0.001).

To understand the effect of the miR-Combo at the level of gene regulation, whole transcriptome analysis was performed in the two PDCs, Ge518 and Ge970.2, where a greater and lower effect on cell viability was found, transfected with the miR-Combo compared to the non-targeting scrambled control (Fig. 3A). After bulk mRNA extraction, RNASeq analysis was performed in three biological replicates for each of the cell lines. In Ge518 PDCs, the gene ontology enrichment analysis revealed expression of genes involved in three main families: cellular and biological processes (*MT2A*, *CDKL5*, *CXCR4*, *MAPK14*, *PLXNA4*, *RAB21*, *TGFBR3*, *VGLL4*), cation transport and homeostasis (*CHRNB2*, *RRAD*, *CACNA1H*, *GPR35*, *P2RX5*, *ATP2A1*), and regulation of growth (*MAPK11*, *NRCAM*, *SHTN1*, *SMURF1*, *CDKN1B*, *TNKS2*, *GDF5*, *VGLL4*) (Fig. 3A). The analysis of miRTarBase, a database of miRNA-target interactions, via g:Profiler [22], showed an enrichment for miR-340-5p (*ROCK1*, *LIMS1*, *ANKRD40*, *SKP2*, *FRS2*) and miR-9-5p (*SFT2D2*, *DICER1*, *IGF2BP3*, *SPON2*, *CXCR4*, *SERPINH1*, *RAP2A*) target genes. Remarkably, several of these target genes were down-regulated following miR-Combo treatment (Fig. 3B, C), and have been shown to be involved in GBM progression (*RAP2A*) [23], angiogenesis (*SPON2*) [24], migration (*CXCR4*) [25], and are associated with an increase in tumor grade (*IGF2BP3*, *SERPINH1*) [26, 27]. In Ge970.2 PDCs, a strong and significant enrichment for genes involved in the antiviral response and type I interferon signaling was found (*OAS-1*, *-2*, *-3*, *-L*, *MX-1*, *-2*, *IFI44L*) (Fig. 3A). Moreover, genes involved in metabolic reprogramming and the pentose phosphatase pathway (TKT, *G6PD*, *PGD*, *TALDO1*) were enriched, as well as NRF2 pathway genes (*NFE2L2*, *GCLC*, *NQO1*, *YES1*), and genes involved in oxidative stress. Altogether, this analysis revealed different cellular responses to miR-Combo in the two models. To identify common hits, we compared the transcriptional profiling of both PDCs, Ge518 and Ge970.2, and found 54 common genes (Fig. 3B). The hierarchical clustering data grouped the samples by cell line first, confirming the significant level of heterogeneity of GBM lines, as, we and others have already described (Fig. 3C) [28–30]. Then, the data were grouped by conditions, miR-Combo vs miR-Ctrl. For both PDCs, we observed a significant decrease of several genes already found to be involved in GBM aggressiveness such as ROCK1 [18, 31], *LIMS1* [18], *SKP2* [32], *NFE2L2* [33], *RAB21* [34]. In contrast, the expression of *ELAVL2*, *RAD9B* and *CNOT3*, already identified as tumor suppressor [35], and inhibitors of cell cycle progression [36], respectively, were upregulated by the miR-Combo (Fig. 3C). Finally, the gene ontology enrichment analysis identified an enrichment for several miRNAs such as miR-340-5p and miR-4255 (*KLHL15*, *LRRC58*, *OTUD4*, *SEC23A*, *SUCO*) (Fig. 3D). Collectively, our data highlighted a subset of critical genes that are modulated in GBM PDCs following miRCombo transfection.

Then, to investigate the effect of each miRNA separately, whole transcriptome analysis was performed in the Ge518 cell line transfected with miR-17-3p and miR-340 mimics and miR-222 antagomir. The gene ontology enrichment analysis revealed expression of genes involved in cellular response to stress as a common pathway for miR-17-3p and miR-222 (Supplementary Fig. 6A). Moreover, we found genes involved in signal transduction for miR-340 and miR-222. As previously shown for miR-340 in a different cellular context [18, 19], we confirmed ROCK1 and LIMS1 as miR-340 target genes (Supplementary Fig. 6A, B).

Moreover, we detected genes involved in neuron development and differentiation, small GTPase mediated signal transduction as well as cell surface receptor signaling pathway. For miR-17-3p, we found three gene families involved in morphogenesis and embryo development, cellular response to stress and response to organonitrogen compound (Supplementary Fig. 6A, C). For miR-222, multiple families of genes were found to be dysregulated such as response to oxidative stress/reactive oxygen species/drug, hematopoiesis and immune system development, and regulation of transcription by RNA polymerase (Supplementary Fig. 6A, D). To confirm these findings, a set of representative genes from each functional family was selected and was validated by quantitative RT-PCR and/or western blots (Supplementary Fig. 6E, F). Furthermore, the expression of several of these genes, such as *NFE2L2*, *COL5A3* and *BDKRB2* for miR-340, *ZFP36*, *NFKBIZ*, and *NTN1* for miR-222, and *LITAF*, and *F2RL2* for miR-17-3p is correlated with poor survival (Supplementary Tables 4 and 5). Of note, when we compared the RNASeq analysis with the predicted miRNA target genes, we found a relatively short number of common hits (Supplementary Fig. 7A). The RNASeq of the miR-Combo *vs* miR-Control (miR-Ctrl) compared with the RNASeq performed for each miRNA separately showed that each miRNA contributes to the gene modulation mediated by the combinatorial strategy (Supplementary Fig. 7B).

To validate miRNA targeting of the 3’UTR, luciferase reporter constructs bearing the full length of E2F-3’UTR and TNFRSF-3’UTR were used and transiently co-transfected in the Ge904 model with the miR-Ctrl vs miR-17-3p and miR-340, respectively (Supplementary Fig. 7C). The ectopic expression of miR-17-3p and miR-340 resulted in a 1.5-fold decrease of luciferase activity in the cells containing the reporter constructs compared to their respective controls, providing evidence that the identified miRNAs act as active miRNAs.

**Fig. 4:**
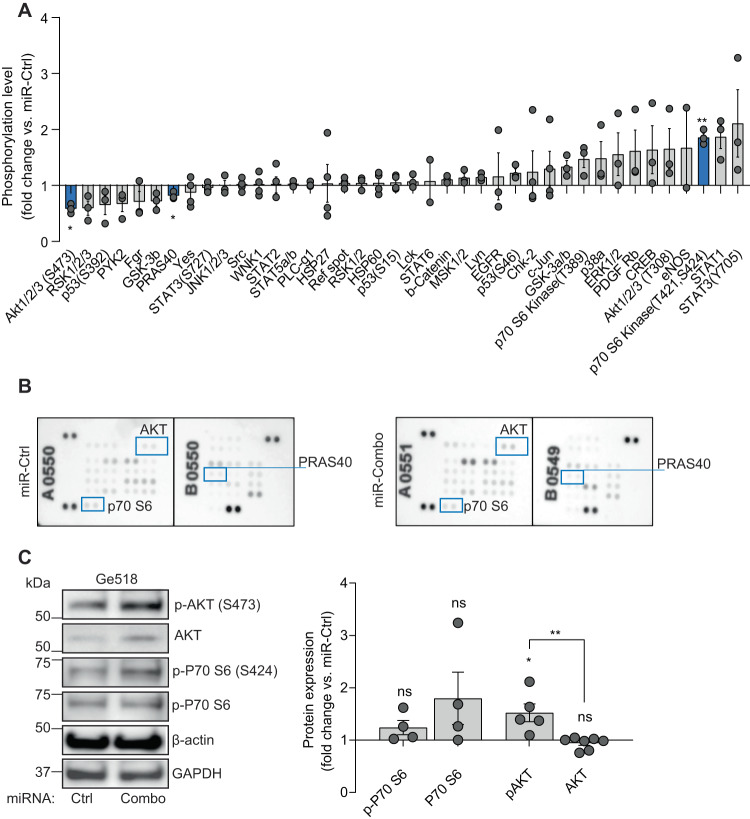
The combinatorial modulation of miR-340, -17-3p and -222 induces a decrease of AKT signaling pathway in GBM PDCs. **A** The human phospho-kinase array shows the relative expression of phosphorylation profiles of several kinases and their protein substrates. Representative pictures of 3 independent experiments. Histograms represent the fold change of each kinase for the miR-Combo versus the miR-Ctrl condition. **B** The human phospho-kinase array shows the relative expression of phosphorylation profiles of several kinases and their protein substrates. Representative pictures of 3 independent experiments. **C** Protein expression was determined by western blot in Ge518 transfected with non-targeting scrambled control or miR-Combo. Histogram represents the fold change of protein expression quantified by using ImageJ (*n* = 4). Data are represented as mean ± SEM (**p < 0.01), ns non-significant.

Mechanistically, to understand how the miR-Combo can modulate downstream signaling, we analyzed the phospho-kinase activity using the human phospho-proteome array (Fig. 4A, B). After transfection, the Ge518 PDCs were submitted to the proteome profiler array, which detects phosphorylated proteins in the cell lysates. We identified a significant increase of p70 S6 kinase and a significant decrease of AKT (S473 residue) and PRAS40 (Fig. 4A). The ribosomal S6 protein kinase, p70 S6 kinase, a downstream substrate of mTOR, is known for its role in controlling cell-cycle progression and cell survival. Moreover, PRAS40 is known to inhibit mammalian target of rapamycin C1 (mTORC1) activity. Indeed, by binding to Raptor, PRAS40 competes with the mTOR substrates, 4E-BP1 and p70S6K. Consistent with the decrease of AKT and PRAS40, we observed a decrease of cell survival in the miR-Combo condition. However, an increase of p70 S6 should be correlated with an improved cell survival. Thus, we evaluated S6 activation, its downstream effector, by western blot analysis and found a significant decrease in the combo condition (Fig. 4C). Moreover, the increase of p70 S6 was associated with an increase of total p70 S6 protein (Fig. 4C). Altogether, our data suggest that the decrease in tumor cell viability might be due to the decrease in the AKT signaling pathway. However, further studies should be performed in order to confirm this conclusion.

### The combinatorial modulation of miR-17-3p, miR-222, and miR-340 inhibits GBM PDC invasion in neural organoids

**Fig. 5:**
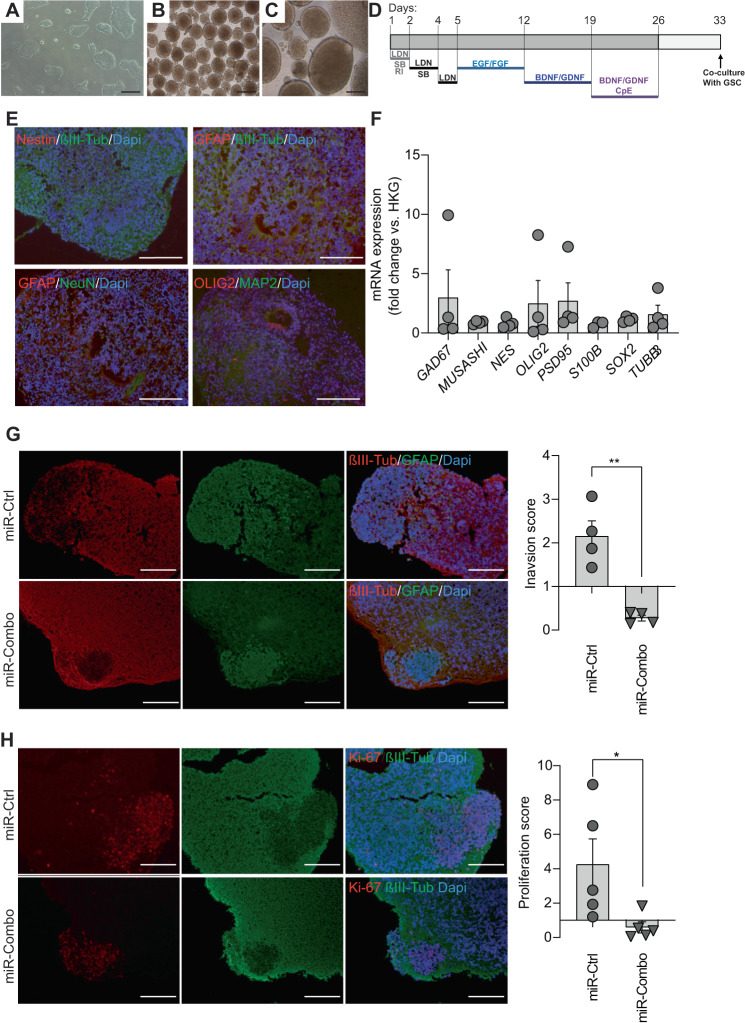
GBM PDC invasion in neural organoids is reduced by the combinatorial modulation of miR-17-3p, miR-222, and miR-340. **A**–**D** Schematic representation of PSC differentiation towards neural organoids. PSC were cultured on Matrigel (**A**) then aggregated in microwell plates (**B**) for 3 weeks (**C**). D Schematic representation of the culture principle for neural organoids. **E** Immunofluorescence shows NeuN, βIII-Tubulin, GFAP, and MAP2-immunoreactive cells present in the neural organoids. Scale bar = 100 µm. **F** GAD67, Musashi, Nestin (NES), OLIG2, PSD95, S100B, SOX2 and βIII-Tubulin (TUBB3) mRNA expression was determined by qPCR in the neural organoids before coculturing with GSC Ge904. Data are normalized to housekeeping genes; mean (*n* = 4) ± SEM. **G** Immunofluorescence shows GFAP, and βIII-Tubulin-immunoreactive cells present in the co-culture of GSC Ge904 and neural organoids. Scale bar = 100 µm. Histograms represent the invasive score of the GBM cells within the neural organoid. **H** Immunofluorescence shows Ki-67, and βIII-Tubulin-immunoreactive cells present in the neural organoids. Scale bar = 100 µm. Histograms represent the proliferative score of the GBM cells within the neural organoid. Data are represented as mean ± SEM (*p < 0.05, and **p < 0.01). Representative images of three separate experiments are shown.

We have recently developed an in vitro tissue engineering approach to generate 3D human brain-like tissue pluripotent stem cell (PSCs) with differentiation towards the astro-neural fate [18, 37]. Previously, we demonstrated that GBM cells proliferate and develop into brain-like tissues, generating a mixed tissue mimicking some critical and important features of the in vivo host / tumor interaction [18, 38]. Therefore, to study the inhibitory potential of the miRNA combo (miR-Combo) in 3D, we used this protocol to generate neural organoids (Fig. 5A–D). The characterization of the neural organoids showed an expression in neural (βIII-Tubulin (*TUBB3*), MAP2, and NeuN), astrocytic (*GFAP*, *S100B*) and oligodendrocytic (*OLIG2*) markers, at the mRNA and protein levels, confirming their neural maturation (Fig. 5E, F). Because GBM stem cells (GSC) represent the most aggressive and drug resistant cells within the tumor, and display self-renewal and tumor-initiation properties, we decided to use them as a model to confirm the miRNA multi-targeting strategy [39, 40]. We co-cultured GSC Ge904 with a neural organoid for 24 h and thereafter transfected with the miR-Combo (Fig. 5G, H). Four days post-transfection, for GSC Ge904, we assessed cell invasion and proliferation with the respective markers GFAP and Ki-67, and for the neural organoids, we used βIII-Tubulin (Fig. 5G, H). We observed invasion of the GSCs into the neural organoids in the miRCtrl condition while the cells transfected with miR-Combo remained compact and not invasive (Fig. 5G). In the miR-Control condition, the GSCs are bigger as they proliferate whereas in the miR-Combo condition they are smaller as if they stop proliferating (Fig. 5H). Accordingly, we observed a stronger signal for Ki-67, a marker of cell proliferation, in the invasive single cells distant from their primary site. Collectively, these data indicate that miRNAs can penetrate the 3D coculture and affect GBM cell behavior by inhibiting their proliferation and invasive capacity.

### The tumor growth of GBM xenografts is reduced by miR-17-3p, miR-222, and miR-340 combo treatment

**Fig. 6:**
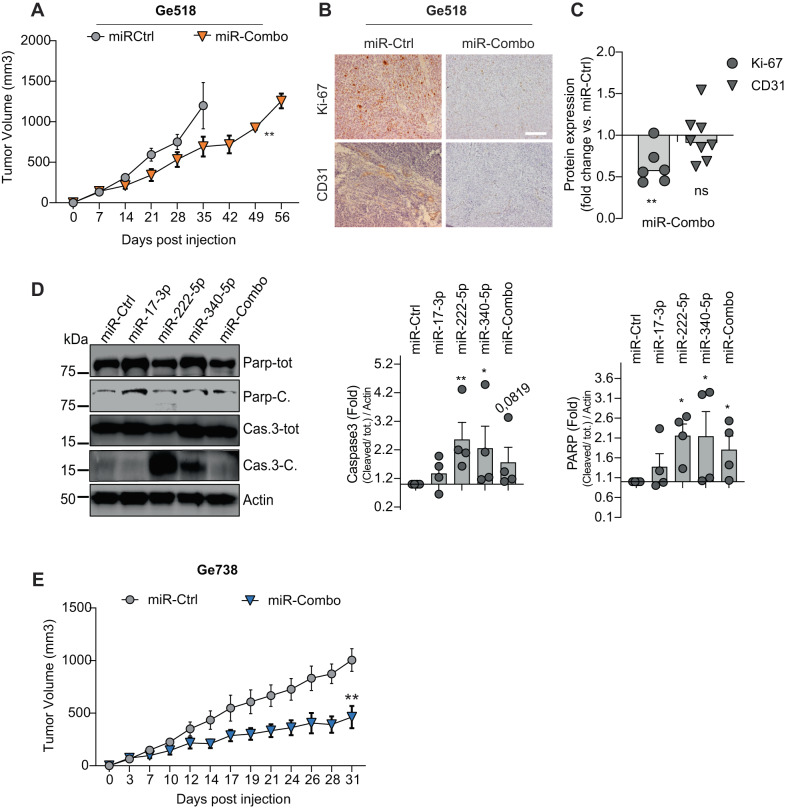
Repeated injection of miR-Combo delays tumor growth in nude mice. **A** Effect of the combinatorial modulation of miR-17-3p, miR-222, and miR-340 on Ge518 tumor growth in vivo (*n* = 5 mice per group). **B** Histological analysis of Ge518 tumor treated with miR-Combo. Tumors were stained for the identified proteins, and counterstained with hematoxylin. Scale bar, 50 µm. **C** Histogram represents the fold change of protein expression quantified by using ImageJ (*n* = 3). Data are represented as mean ± SEM (**p < 0.01), ns non-significant. **D** Protein expression was determined by western blot in Ge518 transfected with non-targeting scrambled control or miR-Combo. Histogram represents the fold change of protein expression quantified by using ImageJ (*n* = 3–4). Data are represented as mean ± SEM (*p  < 0.05, **p < 0.01), ns non-significant. **E** Effect of the combinatorial modulation of miR-17-3p, miR-222, and miR-340 on Ge738 tumor growth in vivo (*n* = 5 mice per group).

To translate our findings to the clinic, we sought to investigate the effects of the combinatorial treatment on Ge518 tumor growth in vivo. To this end, we subcutaneously injected Ge518 cells to the flanks of nude mice (Fig. 6A). Once the tumors reached 150mm^2^, we treated the mice with miR-Combo. We showed a significant delay of tumor growth, indicating that miR-Combo efficiently affects GBM growth and tumorigenic capacity (Fig. 6A), as we have shown in vitro (Fig. 2A). We also found a significant decrease of Ki-67 expression in the miR-Combo compared to the miR-Ctrl (Fig. 6B, C), associated with a significant increase of caspase-3 pathway activation (Fig. 6D), confirming the effect of the combinatorial strategy on GBM cell proliferation. The analysis of tumor vascularization assessed by CD31 staining showed no significant differences but we could observe a trend towards a decrease of CD31 expression and smaller vessels (Fig. 6B, C). To confirm these results, we used another PDC model, Ge738, and showed a similar delay in tumor growth (Fig. 6E). Altogether, we showed that the combinatorial targeting therapy consisting of modulating miR-17-3p, miR-340 and miR-222 efficiently inhibits GBM growth and tumorigenic capacity in vivo.

**Fig. 7:**
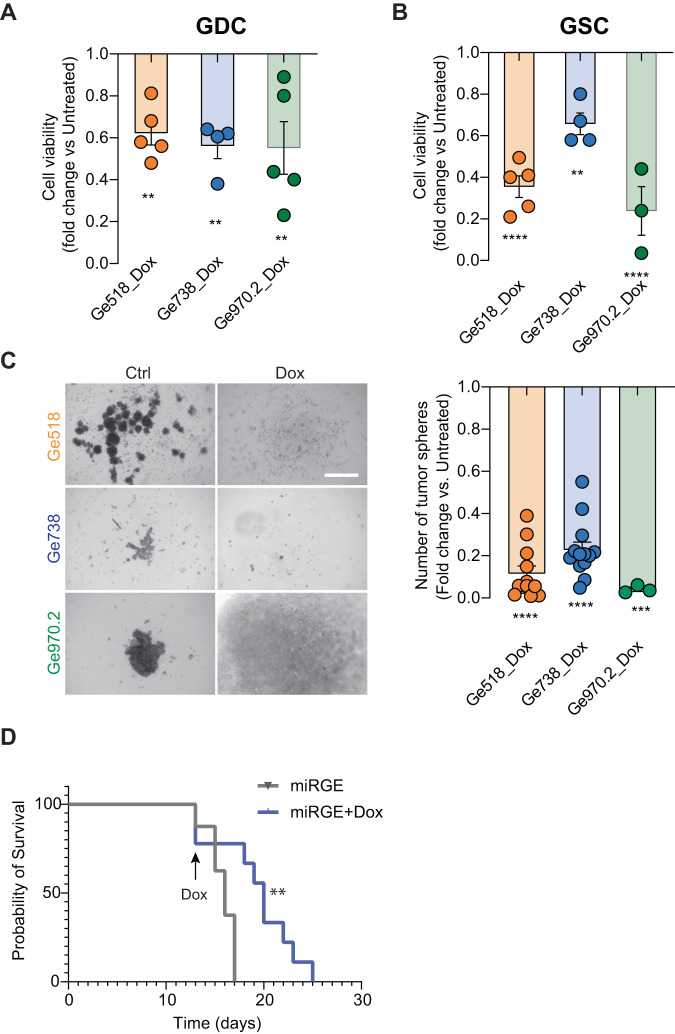
GSC bearing a stable doxycycline-inducible lentivector system expressing miR-17-3p, miR-222, and miR-340 induces a decrease of cell viability and delay tumor growth in vivo. **A** Cell viability of Ge518, Ge738, and Ge970.2 PDCs expressing miRGE was evaluated after three days using CellTiter-Glo. Histograms represent the fold change of cell survival for the miRGE treated with doxycycline (Dox) versus untreated. **B** Cell viability of Ge518, Ge738, and Ge970.2 GSCs expressing miRGE was evaluated after three days using CellTiter-Glo. Histograms represent the fold change of cell survival for the miRGE treated with Dox versus untreated. **C** Representative pictures of 3–7 independent experiments. The bar graph represents the fold change of GSC miRGE treated with Dox versus untreated. Data are represented as mean ± SEM (**p < 0.01, ***p < 0.001 and ****p < 0.0001). **D** Effect of miRGE expression turned on by Dox treatment on tumor growth in vivo: a mixture of Ge518, Ge738, and Ge970.2 GSCs (1:1:1) bearing the inducible Tet-On system was intracranially injected into the brain of nude mice (*n* = 8 mice in the treated group; *n* = 7 mice in the untreated group). Log-rank Mantel–Cox test was used to calculate significance.

By using the well-described miRGE lentivector system [41], we simultaneously expressed miR-17-3p, and miR-340 mimics, and miR-222 antagomiR in GBM PDCs. Then, with the Tet-On system, miRNA modulation was turned on by treating the GBM cells with doxycycline. As shown in Fig. 7A, doxycycline treatment induced a significant decrease of cell viability in all tested PDCs: Ge518, Ge738, and Ge970.2.

Next, we investigated the effect of miRGE on GSCs, an aggressive subset of GBM tumors. Similarly, we observed this effect of doxycycline treatment on miRGE GSC viability after three days, indicating the potential of this combinatorial strategy in both differentiated and stem cells (Fig. 7B). In line with these results, a significant inhibition of their tumorsphere-forming ability was shown under doxycycline treatment (Fig. 7C). More importantly, to evaluate the effect of these miRNAs on GSC tumorigenicity in vivo, we intracranially implanted a mixture of mesenchymal (Ge518), proneural (Ge738) and classical (Ge970.2) GSC transduced with the miRGE lentivector system (1:1:1) into the brain of immunodeficient mice (Fig. 7D). Indeed, a mixture of GSC from several subtypes mimics the in vivo context where multiple subtypes coexist. To leverage our findings to the clinic, the administration of doxycycline to the drinking water was done only after 13 days when the first mice developed neurological symptoms. Here, we showed a significant delay of tumor growth in the doxycycline-treated group compared to the untreated control group (Fig. 7D). Altogether, these results highlight the clinical relevance of the combinatorial strategy as druggable targets for the treatment of GBM, where a significant unmet need for therapies remains.

## Discussion

GBM remains one of the big challenges in cancer therapy for several reasons: high tumor heterogeneity that precludes monotherapy, high invasive capacity giving rise to secondary tumor foci distant to the primary tumor, and resistance to chemo- and radio-therapy leading to recurrence [42]. Hence, the use of a multimodal therapy holds great promise for GBM personalized therapy. miRNAs are small noncoding single-stranded RNAs that regulate several key pathological processes including tumor proliferation, invasion, metastasis or resistance to therapy. By designing targeted therapy to alter miRNA levels, we could specifically modulate their expression to inhibit tumor progression [43].

By performing a comprehensive analysis of the TCGA dataset, we identified four miRNAs important for the overall survival of GBM patients with or without treatment. miR-17-3p, miR-340 and miR-222 are critical miRNAs that modulate GBM cell viability in vitro and in vivo. Indeed, our data provide a proof-of-concept for delivering this combinatorial miRNA therapy based on two mimics and one antagomiR to treat GBM tumors. This proof-of-concept for co-delivery of two miRNAs was previously tested in GBM tumors where they obtained a delay of tumor growth [44, 45]. In comparison, our study proposed a multi targeting system composed of three miRNAs efficient against all three subtypes of GBM tumors, suggesting a broader effect of this combination.

The biological effect with one single miRNA can be very relevant in its magnitude in some subtypes but not in others. For example, in our study we showed that miR-17-3-p affects cell survival in one mesenchymal PDCs (Ge518) but not in classical PDCs (Ge904, Ge970.2). Based on our data, to provide an efficient therapy for all GBM patients, the combination of these three miRNAs is required for a systematic inhibition of tumor growth, regardless of the tumor subtype.

We could not observe a strong biological activity of miR-551b on GBM aggressiveness even though it is a good prognostic predictor and therapeutic target for gastric, ovarian and colon carcinoma [46–48]. Nevertheless, miR-551b remains of interest for its effect on GBM microenvironment and/or chemoresistance mechanisms [49].

miR-222 and miR-221 are highly homologous and often work synergistically. Their expression has been extensively investigated, and they are frequently overexpressed in various cancers including GBM [50, 51]. Thanks to a large number of previous studies, the role of miR-222 is already well characterized in GBM. However, in this study, we only modulated miR-222 and not miR-221, in order to understand the miR-222 contribution in GBM cells as it was identified as a critical miRNA from our TCGA analysis. In GBM, the inhibition of the miR-221/222 cluster induces a decrease in cell proliferation, migration, and an increase in apoptosis and angiogenesis. Several target genes were found to be downregulated by the miR-221/222 cluster such as *MMP-9*, *MMP-2*, *VEGF*, *Ki-67*, *TRAIL*, or the suppressor of cytokine signaling-3 (*SOCS3*) [52–54]. Here, we identified potential molecular targets of miR-222 in patient-derived GBM cells like *ZFP36*, *JUN*, *FOS*, *TNF*, and *CXCL8* as their expression was decreased with miR-222 downregulation. The modulation of ZFP36 expression was shown to impair cell growth and invasive capacity of GBM cells [55, 56]. Similarly, TNF-α stimulation was associated with GBM cell invasion and matrix metalloproteinase-2 (MMP-2) activity [57]. By modulating only miR-222, and not miR-221, this study highlighted the potential downregulated genes mediating its effect on GBM cell fate.

In vitro and in vivo, we found a strong biological activity of miR-17-3p on GBM aggressiveness. miR-17-3p belongs to the miR-17-92 cluster along with miR-17-5p, miR-18a, miR-19a, miR-19b, miR-20a, and miR-92a. Members of this cluster are widely expressed in various cancer types such as lymphoma, lung, colon and breast carcinoma. When we modulated miR17-3p in Ge518, we found several genes involved in the Wnt pathway, NF-Kappa-B activation or p53 signaling. Moreover, we identified *VIM*, *LITAF*, and *IL1A* as targeted genes which have already been associated with various features of GBM aggressiveness [42, 58, 59]. In GBM, expression of miR-17-5p, but not miR-17-3p, was previously reported to be associated with better survival [12]. Few studies have assessed the role of miR-17-3p in GBM aggressiveness and its potential as a therapeutic target [60].

We, and others, have already shown that miR-340 down-expression is correlated with poor survival and its upregulation with an inhibition of cell growth and motility [18]. Moreover, ROCK1 was found to be a target of miR-340 in different GBM cell lines. In this study, we identified miR-340 as one of the four clinically relevant miRNAs in GBM and confirmed ROCK1 as its target. Moreover, we identified *ROCK1*, *NFE2L2*, *LIMS1*, and *TMEM64* as common dysregulated genes modulated by miR-340 in our previous and current studies [18]. Collectively, we found miR-17-3p and miR-340 as two strong regulators of GBM aggressiveness in GBM cells. A more in-depth detailed analysis of both miR-17-3p and miR-340 modulation could be carried out to address this observation. Several previous studies have generated and evaluated a miRNA expression signature to predict survival in GBM [12–14]. When we compared our results with these studies, we were able to find almost all these miRNAs in one or several of our subgroups (before or after treatment), such as miR-222, miR-221, miR-200b, miR-200a, miR-148a, miR-10a and miR-615. By promoting cell proliferation, and invasion through the inhibition of several tumor suppressors genes and pathways, the expression of miR-21 has been associated with the aggressive phenotype cancer cells [61]. Indeed, miR-21 has been identified as an important oncomiR, highly expressed in various cancer types. In our study, we observed a significant correlation between high expression of miR-21 and poor patient survival. However, this holds true only in the subgroup of patients who did not receive any treatment. Therefore, this miRNA was not included in the miR-Combo. Nevertheless, several studies aiming at investigating the effect of this specific miRNA on GBM aggressiveness have already showed its efficiency on cellular and animal models [62, 63]. Similarly, other miRNAs were able to inhibit GBM cell aggressiveness.

When compared to other small molecule therapeutics, mimics and antagomirs represent an attractive and effective strategy as they can inhibit non-druggable targets, such as proteins lacking enzymatic activity or that exhibit inaccessible structural conformations [64]. Indeed, mimics and antagomirs can hit virtually any target and do not require stable expression through plasmid, viral or nanoparticle delivery systems. Moreover, the dosing of mimics and antagomirs can be adjusted along treatment regimen or withdrawn (in case of toxicity or off-target effects inducing adverse reactions) [65]. In this study, we used an intraperitoneal delivery of the mimic miR-17-3p and miR-340 along with the antagomir miR-222. Even if we observed a significant delay of tumor growth in all conditions, the relative merits of intravenous and subcutaneous delivery may be the subject of further research to achieve higher efficiency. Moreover, the sequential injection of each mimic and antagomir separately and not concomitantly could be performed to avoid the occurrence of a mismatching event and to obtain a better outcome. Moreover, the use of miR-222 antagomir could be combined with miR-221 antagomir to optimize inhibition of tumor growth. For all these reasons, the combo seems to have a less inhibitory effect in vivo, while significant, compared to in vitro. Nevertheless, this study showed a proof of concept for using the combinatorial delivery of miRNAs to treat GBM tumor xenografts in vivo.

The LNA-modified anti-miR-122, known as Miravirsen was the first miRNA-targeting therapeutic to enter clinical trials. Because miR-122 is critical for hepatitis C virus (HCV) RNA stabilization, Miravirsen was intended for HCV-infected patients. Consequently, Miravirsen blocks HCV multiplicity capacity and has shown good results in the management of HCV.

Regarding miRNA-based cancer therapy, the MRX34, a miR-34a mimic enclosed in liposomes, entered a phase I clinical study and showed stable disease (six patients) or prolonged partial response (one patient) (https://clinicaltrials.gov/show/NCT01829971). Unfortunately, even if the clinical responses were encouraging, this trial was terminated early due to severe immunerelated events. TargomiRs, made from a consensus sequence of several members of the miR-15/16 family, were shown to be efficient on malignant pleural mesothelioma and non-small cell lung cancer patients (https://clinicaltrials.gov/show/NCT02369198) [66]. Finally, the LNA-modified anti-miR-155, known as MRG-106 (cobomarsen), was initiated for patients with cutaneous T cell lymphoma (https://clinicaltrials.gov/show/NCT02580552) [67].

Considering the number of preclinical studies and clinical trials, miRNA-based cancer therapy opens up a new avenue in cancer management. To increase miRNA stability and effective delivery, several carriers (viral and non-viral based) and/or chemical modifications can be used. However, even if these different strategies succeed in tackling the different issues that derive from miRNA-based cancer therapy, the systemic toxicity along with the immunogenicity response remains challenging. In this study, we mainly used miRNA mimics to rescue miRNA expression in tumor cells. We believe that this approach may decrease or circumvent the severe side effects previously observed by using antagomirs, which aim at inhibiting miRNA expression.

Altogether, our study participated in this effort and validated a miRNA combinatorial therapeutic strategy that can be developed and used for the treatment of GBM patients. Hopefully, these strategies will help prolong patient survival and increase their quality of life in the future.

## Materials and methods

### GBM cell lines and patient-derived models

Eight GSCs from different subtypes Ge269, 518, 835, (mesenchymal subtype) 738, 898, (proneural subtype) 885, (neural subtype) 904, 970.2 (classical subtype) were cultured in DMEM/F12 with Glutamax supplemented with B27 supplement and b-FGF, EGF both at 10 ng/ml and 1% penicillin/streptomycin, as previously described [18]. Of note, Ge970.2 is a PDC line with a low rate of growth which precludes its use for all readouts. To generate GBM PDCs, we transferred the GSCs to, and maintained them in, Dulbecco’s modified Eagle’s medium (DMEM)-high glucose/glutamax, 10% fetal bovine serum (FBS), and 1% penicillin/streptomycin (GDC medium). All PDC lines were validated to be mycoplasma negative before experiments.

### Chemicals

Actinomycin D and Doxycycline were obtained by Sigma and used at the concentration of 5 µM and 1 mg/ml for 24 h, respectively.

### Cell transfection

The miR-17-3p, miR-340-5p and miR-551b-5p mimics, and miR-222-5p antagomir were transfected using lipofectamine RNAimax (Invitrogen), at a final concentration of 5 nM, according to the manufacturer’s protocols. A non-targeting scrambled miRNA (Life Technologies) was used as control.

### Cell viability assay

Cell viability assay was performed by using CellTiter-Glo assay kit (Promega) as previously described [18, 21]. For more details, see Supplementary Information.

### Cell apoptosis and necrosis assay

These experiments were performed by using RealTime-Glo Annexin V Apoptosis and Necrosis Assay (Promega) according to the manufacturer’s protocols. Also, 96-well plates were used to seed 1000 cells after transfection. Fluorescence and luminescence were used to measure cell apoptosis and/or necrosis.

### AlexaFluor 488 Annexin V/PI dead cell apoptosis kit

Day 3 post-transfection, patient-derived GDC were subjected to Annexin V/PI dead cell apoptosis assay according to the manufacturer’s protocols. Briefly, after resuspending the cells in 1× annexin-binding buffer, they were stained with Annexin V/PI working solution during 15 min in the dark prior to flow cytometric analysis carried out using FACS Fortessa and data were processed using FlowJo software.

### Cell transmigration

Cells (2 × 10^4^) were seeded onto the filters of transwell polycarbonate membrane inserts (24 well, 8 µm pores, Corning) in serum-free (0% FBS) GDC medium and the lower compartments were filled with 10% FBS GDC medium. After 24 h of incubation at 37 °C to allow transmigration assessment, the adherent cells on the lower surface were stained with 0.1% crystal violet and quantified and/or counted. Images were captured using EVOS microscope (Life Technologies) and manually counted.

### Soft agar assay

In all, 48-well plates were used to seed 5000 cells in 0.3% agar/GDC medium on top of a bottom layer of 1% agar. In all, 200 µl of additional GDC medium was added on top and cells were cultured for at least 15 days. Clonogenic assay was determined by counting colonies stained with with 0.1% crystal violet/20% methanol/PBS. Each colony is considered to be a minimum of 50 cells.

### miRGE lentivector

miRGE construct bearing miR-17-3p and miR-340-5p mimics and miR-222-5p antagomir was generated by Vector Lab (Dr. Patrick Salmon, University of Geneva), as previously reported [41].

### Luciferase assay

At day 0 (D0), PDCs Ge904, chosen because they do not express miR-340 and miR-17-3p, were seeded at 80,000 cells per well in a 24-well plate. Twenty-four hours later at D1, cells were transfected with the corresponding miRNA and plasmid using Lipofectamine 3000 according to the manufacturer’s protocol (L3000-008 Invitrogen). Twenty-four hours later at D2, cells were lysed in the wells using the passive Lysis buffer from the Dual Luciferase Reporter Assay (E1910 Promega), followed by a measurement of the luminescence according to the manufacturer’s protocol.

Results were normalized to Renilla control.

### Neural organoids

Human induced PSC line (iPSCs) and embryonic stem cells (ESCs) were used to generate the neural organoids, as previously described with minor modifications [37]. iPSCs was kindly provided by Dr. Youssef Hibaoui and generated as previously described [68]. The human ESCs, HS420, was kindly provided by the Professor Karl-Heinz Krause. For more details, see Supplementary Information.

### 3D invasion and proliferation score

Four days post-transfection, cell invasion score was measured according to two different parameters: the distance between the implantation site (named primary site) and the distant secondary site, when available on the same section, as well as the dispersion of the cancer cells with the organoid. Images were captured using Nikon Eclipse C1 Confocal microscope as well as a Nikon Eclipse TE2000-E and analyzed using the “ROI” feature in ImageJ. For the proliferation score, KI67 staining was quantified with ImageJ on three different slides for each separate experiment.

### Human phospho-kinase assay

The phosphorylation profiles of kinases were performed using the human phospho-kinase array kit (R&D Systems) according to the manufacturer’s recommendations. For quantification, dots were analyzed on Image J software using the analyze gels, plot lane command. All dots were normalized to the negative control. Combo conditions were normalized to the control conditions.

### Immunoblotting

Proteins were extracted in IP-MS cell lysis buffer (Life Technologies) and quantified using the Pierce BCA kit (Thermo Fisher) as previously described [21]. The following antibodies were used for immunoblotting: Vimentin (Millipore), p-P70 S6 Kinase (Cell Signaling), P70 S6 Kinase (Cell Signaling), pS6 (Cell Signaling), S6 (Cell Signaling), Cleaved-Caspase 3 (Cell Signaling), Caspase 3 (Cell Signaling), Cleaved-PARP (Cell Signaling), PARP (Cell Signaling), GAPDH (Cell Signaling), LITAF (Santa Cruz Biotechnology), and β-actin HRP (Sigma-Aldrich) as loading control. For protein expression analysis, expression was normalized to β-actin (and/or the total protein for phospho-proteins) then compared to their respective control.

### Immunohistochemistry (IHC) and immunofluorescence (IF)

IHC and IF staining of formalin-fixed paraffin-embedded tissues was carried out as previously described [18, 63]. Sections were then incubated overnight at 4 °C with primary antibody pAKT (Cell Signaling), AKT (Cell Signaling), Ki-67 (Chemicon), and CD31 (Abcam) followed by, for IHC, biotin-conjugated anti-rabbit IgG and an avidin–biotin peroxidase detection system with 3,3’-diaminobenzidine substrate (Vector), then counterstained with hematoxylin (Sigma).

For IF, sections were then incubated overnight at 4 °C with primary antibody βIII-Tubulin (Covance), GFAP (Dako), MAP-2 (Millipore), NeuN (Millipore), and Ki-67 (Chemicon). Then, fluorochrome-labeled secondary antibodies were used: Alexa Fluor (555 and/or 488)-labeled antibodies from goat or donkey against mouse, goat, or rabbit (Molecular Probes). Images were captured using a Zeiss AxioCam microscope. Data were analyzed using the Threshold Color plugin feature in ImageJ (National Institutes of Health).

### Reverse transcription quantitative PCR (RT-qPCR)

Isolation of total RNA was carried out by using RNeasy kit from Qiagen according to the manufacturer’s instructions and as previously described [18]. Primer sequences are described in Supplementary Table 1. Prior to utilization, an efficacy test was performed, and all primers were validated. At least, two housekeeping genes (EEF1A1 and ALAS1) were used for normalization. RT-PCR reactions were performed in, at least, three technical and biological triplicates, and the average cycle threshold (CT) values were determined. For miRNAs, miR16-5p, and miR-191-5p were validated and used as housekeeping genes. For more details, see Supplementary Information.

### Analysis of RNASeq data

As previously described [18], total RNA was extracted using the Trizol method. Then, after checking RNA quality, the SR100 – libraries TruSeqHT stranded – Illumina HiSeq 4000 was used and the sequencing quality control was done with FastQC v.0.11.5. The quality distribution along the reads plot validated for all samples. The reads were mapped with STAR aligner v.2.5.3a to the UCSC human hg38 reference. The average mapping rate was 92.97%. The differential expression analysis was performed with the statistical analysis R/Bioconductor package edgeR v. 3.18.1 60. Briefly, the counts were normalized according to the library size and filtered. The genes having a count above one count per million reads (cpm) in at least four samples were kept for the analysis. The raw gene number of the set is 26,485. The poorly or not expressed genes were filtered out. The filtered data set consists of 12,737 genes. The differentially expressed genes tests were done with exact Test using a negative binomial distribution. The differentially expressed genes *p* values are corrected for multiple testing error with a 5% FDR (false discovery rate). The correction used is Benjamini–Hochberg. Then, Fig. 3C was generated through Morpheus (https://software.broadinstitute.org/morpheus).

### In silico data analysis

*p* values for Kaplan–Meier log-rank-test analysis of target genes were done by using R software and Python (rpy2). We compared survival curves by log-rank test, the endpoint being overall survival. All individuals were classified by cutoff (sd above and below the mean as well as the median). The gene enrichment analysis was done using g:Profiler and DAVID Bioinformatics resources [22, 69, 70].

### TCGA analysis

miRNA expression data and the corresponding clinical data for GBM samples were downloaded (http://cancergenome.nih.gov/) from the TCGA data portal and analyzed as previously described [18]. At the time of this analysis, 526 miRNAs were analyzed in 565 patients.

### Subcutaneous injection

All animal procedures were performed in accordance with the ethics committee for animal research of Geneva under the approved protocol GE/38/20 according to the standards set by the institutional animal care. Six- to 10-week-old female nu/nu immunocompromised mice weighing 20–25 g (Charles River Labs) were housed five per cage, with standard husbandry for specific pathogen free (SPF) provided by animal facility staff. Prior experimentation, mice were allowed to acclimate for 2 weeks. Ge518, and Ge738 GSCs (5 × 10^6^ tumor cells in 200 µl of PBS) were injected subcutaneously into the right flank of mice. Tumor sizes were monitored three times per week with caliper until they reached a size of 150 mm^3^. Animals were randomly allocated to experimental groups. The investigator was blinded to the group allocation but not when assessing the outcome.

### Dosing

The mimic-invivofectamine and antagomir-invivofectamine complex was prepared according to the manufacturer’s instructions. The final concentration of mimic/antagomir was 1.75 mg/ml per 200 µl per mouse (corresponding to 5 nmol). As a first pulse, mice were injected twice with the aforementioned concentration, then every other day with 0.875 mg/ml per 200 µl per mouse (corresponding to 2.5 nmol). The solution was maintained at room temperature until the intraperitoneal injection. The mirVana^TM^ negative control mimic was used for the control group. For the mimics, we used mirVana^TM^ miR-17-3p (MC12246), mirVana^TM^ miR-340-5p (MC12670), and for the antagomir mirVana^TM^ miR-222 (MH11376).

### Orthotopic brain tumor injection

Ge518, Ge738, and Ge970.2 bearing miRGE were orthotopically transplanted following washing and resuspension in PBS into 6–10-week-old female nu/nu immunocompromised mice. Briefly, a Hamilton syringe mounted on the frame was used to inject tumor cells (1 μl/min) into the putamen (through the preformed hole to a depth of 3.6 mm, a site far from the ventricles, and with little critical activity in the mouse). The withdrawal of the syringe was done over 5 min and the hole and scalp were plugged and closed with bone wax and two sutures, respectively. Body weight was measured twice weekly for the duration of the experiments and prior to injection. 15% of the pre-procedure body weight led to euthanasia. To assess tumor progression, animals were monitored daily and those exhibiting signs of morbidity and/or development of neurological symptoms were euthanized immediately. With this Tet-on system, miRNA expression can be turned on by treating the mice with doxycycline used at 0.5 mg/ml, which is given in the drinking water and replaced every 2 days. Animals were randomly allocated to experimental groups. The investigator was blinded to the group allocation but not when assessing the outcome.

### Quantification and statistical analysis

Sample size and statistics for each experiment are provided in the Results section and figure legends. Data shown are representative of results obtained for multiple experiments as noted in the figure legends. All statistical analyses were performed using one-way analysis of variance (ANOVA) and Student’s *t* test, with *p* < 0.05 considered significant. All statistical analyses were carried out using Prism software (GraphPad). Chi-squared tests or *t* tests were used to calculate statistical significance.

### Supplementary information

Figure S1

Figure S2

Figure S3

Figure S4

Figure S5

Figure S6

Figure S7

Table S1

Table S2

Table S3

Table S4

Table S5

Supplementary Legends.

Original Data File

Reproducibility checklist

## Data Availability

Further information and requests for resources and reagents should be directed to and will be fulfilled by the lead contact and corresponding author, upon reasonable request. All RNASeq raw data will be deposited to Gene Expression Omnibus upon paper acceptance.
