# Supplementary material for: Identification of a miRNA multi-targeting therapeutic strategy in glioblastoma
Source: Cell Death Dis. 2023 Sep 25;14(9):630. doi: 10.1038/s41419-023-06117-z (PMC10519979; doi:10.1038/s41419-023-06117-z)
Supplement: Supplementary file 8 — Table S1 [file 41419_2023_6117_MOESM8_ESM.docx]

Supplementary Table S1

| **Gene** | | | **Forward** | | | | **Reverse** | | | |
| --- | --- | --- | --- | --- | --- | --- | --- | --- | --- | --- |
| **VIM** | | | GACGCCATCAACACCGAGTT | | | | CTTTGTCGTTGGTTAGCTGGT | | | |
| **RAP2A** | | | CGTGAAGCGGTATGAGAAAGT | | | | GCCTTCGCTGGACGATACT | | | |
| **LITAF** | | | ATGTCGGTTCCAGGACCTTAC | | | | TACGAAGGAGGATTCATGCCC | | | |
| **NFKBIZ** | | | ACACCCACAAACCAACTCTGG | | | | GGCAAAACTGTGATTCTGGACC | | | |
| **ROCK1** | | | AACCATGTGACTGAGTGCCC | | | | TCAGTGTGTTGTGCCAAAGC | | | |
| **CHRNA4** | | | GGAGGGCGTCCAGTACATTG | | | | GAAGATGCGGTCGATGACCA | | | |
| **BDKRB2** | | | GTCTGTTCGTGAGGACTCCG | | | | AAAGGTCCCGTTAAGAGTGGG | | | |
| **GALNT3** | | | TCTTGGACCAGACACTCGAC | | | | GGACAGTTCTAAGCAACGTGG | | | |
| **STMN3** | | | CGCACCCCAATACCGTCTAC | | | | GGACAGGTCAGAAGGGGACTT | | | |
| **COL5A3** | | | GTGGCCGTCAGCATAGATGG | | | | TGAATGTCTCCCTCGAAAGTCTT | | | |
| **NES** | | | GGAAGAGAACCTGGGAAAGG | | | | CTTGGTCCTTCTCCACCGTA | | | |
| **OLIG2** | | | GGACAAGCTAGGAGGCAGTG | | | | ATGGCGATGTTGAGGTCGTG | | | |
| **PSD95** | | | ACAAGCGGATCACAGAGGAG | | | | CAGATGTAGGGGCCTGAGAG | | | |
| **S100B** | | | CATCGACGTTTTCCACCAATA | | | | TCGTGGCAGCGAGTAGTAAC | | | |
| **SOX2** | | | GCGAACCATCTCTGTGGTCT | | | | GGAAAGTTGGGATCGAACAA | | | |
| **TUBB3** | | | CGGTGGTGGAACCCTACAAC | | | | AGGTGGTGACTCCGCTCAT | | | |
| **EEF1A1** | | | AGCAAAAATGACCCACCAATG | | | | GGCCTGGATGGTTCAGGATA | | | |
| **ALAS1** | | | CTCACCACACACCCCAGATG | | | | AGTTCCAGCCCCACTTGCT | | | |
|  |  |  |  |  |  |  |  |  |  |  |
| **miRNAs** | | | **Sequence** | | | |  |  |  |  |
| **miR-340-5p** | | | UUAUAAAGCAAUGAGACUGAUU | | | |  |  |  |  |
| **miR-17-3p** | | | ACUGCAGUGAAGGCACUUGUAG | | | |  |  |  |  |
| **miR-222-3p** | | | AGCUACAUCUGGCUACUGGGU | | | |  |  |  |  |
| **miR-222-5p** | | | CUCAGUAGCCAGUGUAGAUCCU | | | |  |  |  |  |
| **miR-551b** | | | GAAAUCAAGCGUGGGUGAGACC | | | |  |  |  |  |
| **miR-16-5p** | | | UAGCAGCACGUAAAUAUUGGCG | | | |  |  |  |  |
| **miR-191-5p** | | | CAACGGAAUCCCAAAAGCAGCUG | | | |  |  |  |  |
