# Supplementary material for: Identification of a miRNA multi-targeting therapeutic strategy in glioblastoma
Source: Cell Death Dis. 2023 Sep 25;14(9):630. doi: 10.1038/s41419-023-06117-z (PMC10519979; doi:10.1038/s41419-023-06117-z)
Supplement: Supplementary file 9 — Table S2 [file 41419_2023_6117_MOESM9_ESM.docx]

Supplementary Table S2

| **All** | | | | **NO Treatment** | | | **WITH Treatment** | | |  |  |  |
| --- | --- | --- | --- | --- | --- | --- | --- | --- | --- | --- | --- | --- |
|  | **Risk factor** | | |  | **Risk factor** | |  | **Risk factor** | |  |  |  |
| **name** | **Positive** | **Negative** | | **name** | **Positive** | **Negative** | **name** | **Positive** | **Negative** |  |  |  |
| hsa-miR-34a |  |  | | hsa-miR-18a* |  |  | hsa-miR-17-3p |  |  |  |  |  |
| hsa-miR-218 |  |  | | hsa-miR-551a |  |  | hsa-miR-200b |  |  |  |  |  |
| hsa-miR-196a |  |  | | hsa-miR-609 |  |  | hsa-miR-155 |  |  |  |  |  |
| **hsa-miR-340** |  |  | | hsa-miR-302c |  |  | hsa-miR-218 |  |  |  |  |  |
| **hsa-miR-222** |  |  | | hsa-miR-483 |  |  | hsa-miR-196a |  |  |  |  |  |
| hsa-miR-200b |  |  | | hsa-miR-767-5p |  |  | hsa-miR-361 |  |  |  |  |  |
| hsa-miR-221 |  |  | | hsa-miR-514 |  |  | hsa-miR-140 |  |  |  |  |  |
| **hsa-miR-17-3p** |  |  | | hsa-miR-615 |  |  | hsa-miR-34a |  |  |  |  |  |
| hsa-miR-155 |  |  | | ebv-miR-BART14-5p |  |  | hsa-miR-92 |  |  |  |  |  |
| hsa-miR-148a |  |  | | hsa-miR-206 |  |  | hsa-miR-199b |  |  |  |  |  |
| kshv-miR-K12-2 |  |  | | hsa-miR-647 |  |  | **hsa-miR-340** |  |  |  |  |  |
| hsa-miR-551b |  |  | | hsa-miR-663 |  |  | hsa-miR-148a |  |  |  |  |  |
| hsa-miR-101 |  |  | | hsa-miR-571 |  |  | hsa-miR-191 |  |  |  |  |  |
| hsa-miR-10a |  |  | | hsa-miR-453 |  |  | hsa-miR-222 |  |  |  |  |  |
| hsa-miR-7 |  |  | | hcmv-miR-UL70-5p |  |  | hsa-miR-10a |  |  |  |  |  |
| hsa-miR-140 |  |  | | hsa-miR-199a |  |  | hsa-miR-101 |  |  |  |  |  |
| hsa-miR-124a |  |  | | hsa-miR-21 |  |  | hsa-miR-494 |  |  |  |  |  |
| hsa-miR-200a |  |  | | hsa-miR-105 |  |  | hsa-miR-498 |  |  |  |  |  |
| hsa-miR-196b |  |  | | hsa-miR-657 |  |  | hsa-miR-363 |  |  |  |  |  |
| hsa-miR-34c |  |  | | hsa-miR-515-3p |  |  | hsa-miR-409-3p |  |  |  |  |  |
| hsa-miR-30e-3p |  |  | | hsa-miR-524* |  |  | hsa-miR-335 |  |  |  |  |  |
| hsa-miR-92 |  |  | | hsa-miR-583 |  |  | hsa-miR-197 |  |  |  |  |  |
| hsa-miR-487b |  |  | | hsa-miR-222 |  |  | hsa-miR-551b |  |  |  |  |  |
| hsa-miR-34b |  |  | | hsa-miR-623 |  |  | hsa-miR-196b |  |  |  |  |  |
| hsa-miR-133a |  |  | | ebv-miR-BART2 |  |  | hsa-miR-30e-3p |  |  |  |  |  |
| hsa-miR-199b |  |  | | hsa-miR-551b |  |  | hsa-miR-522 |  |  |  |  |  |
| hcmv-miR-UL70-5p |  |  | | hsa-miR-539 |  |  | hsa-miR-520e |  |  |  |  |  |
| hsa-miR-526c |  |  | | hsa-miR-505 |  |  | TOTAL | **13** | **14** |  |  |  |
| sa-miR-204 |  |  | | hsa-miR-642 |  |  |  |  |  | | |  |
| hsa-miR-30e-5p |  |  | | hsa-miR-137 |  |  |  |  |  | | |  |
| hsa-miR-566 |  |  | | kshv-miR-K12-4-5p |  |  |  |  |  | | |  |
| TOTAL | **10** | **21** | | hsa-miR-638 |  |  |  |  |  | | |  |
|  |  |  |  | hsa-miR-565 |  |  |  |  |  | |  |  |
|  |  |  |  | hsa-miR-661 |  |  |  |  |  | |  |  |
|  |  |  |  | hsa-miR-17-3p |  |  |  |  |  | |  |  |
|  |  |  |  | kshv-miR-K12-6-5p |  |  |  |  |  | |  |  |
|  |  |  |  | hsa-miR-767-3p |  |  |  |  |  | |  |  |
|  |  |  |  | hsa-miR-15b |  |  |  |  |  | |  |  |
|  |  |  |  | hsa-miR-520d* |  |  |  |  |  | |  |  |
|  |  |  |  | **hsa-miR-340** |  |  |  |  |  | |  |  |
|  |  |  |  | TOTAL | **26** | **14** |  |  |  | |  |  |
