# Supplementary material for: Identification of a miRNA multi-targeting therapeutic strategy in glioblastoma
Source: Cell Death Dis. 2023 Sep 25;14(9):630. doi: 10.1038/s41419-023-06117-z (PMC10519979; doi:10.1038/s41419-023-06117-z)
Supplement: Supplementary file 10 — Table S3 [file 41419_2023_6117_MOESM10_ESM.docx]

Supplementary Table S3

|  | **mir340-**  **3p** | **mir340-**  **5p** | **mir551b-**  **5p** | **mir551-**  **3p** | **mir17-3p** | **mir17-5p** | **mir222-**  **3p** | **mir222-**  **5p** |  |  |
| --- | --- | --- | --- | --- | --- | --- | --- | --- | --- | --- |
| **269** | ++ | ++ |  |  |  | + |  |  | Mes |  |
| **518** |  |  |  |  | + | + |  | +++ | Mes |  |
| **688** |  | + |  |  |  | + |  | + | Clas |  |
| **738** | + | + |  |  | +++ | +++ |  | +++ | Pron |  |
| **885** | + |  |  |  |  |  |  |  | Neu |  |
| **898** | + | + |  |  |  |  |  |  | Pron |  |
| **970.2** | + |  |  |  |  |  |  |  | Clas |  |
| **835** |  |  |  |  |  |  | ++ |  | Mes |  |
| **904** |  |  |  | + |  |  |  |  | Clas |  |
| **U87** |  |  |  |  |  | + | + |  | UD |  |
| **U251** |  |  |  |  |  |  | + |  | UD |  |
