# Supplementary material for: Identification of a miRNA multi-targeting therapeutic strategy in glioblastoma
Source: Cell Death Dis. 2023 Sep 25;14(9):630. doi: 10.1038/s41419-023-06117-z (PMC10519979; doi:10.1038/s41419-023-06117-z)
Supplement: Supplementary file 11 — Table S4 [file 41419_2023_6117_MOESM11_ESM.docx]

Supplementary Table S4

|  | *Log-rank pValue* | | |
| --- | --- | --- | --- |
| **Name** | **TCGA** | **Rembrandt** | **Gravendeel** |
| **NFE2L2** | 0,0015 | 0,0064 | 0,0109 |
| **COL5A3** | 0,0757 | <0,01 | 0,0005 |
| **ZFP36** | 0,0974 | 0,0162 | 0,0006 |
| **NFKBIZ** | 0,0637 | <0,01 | <0,01 |
| **SHISA9** | 0,0232 | 0,0013 | 9,77E-02 |
| **NTN1** | 0,0077 | 0,2524 | 0,6112 |
| **LITAF** | 0,0032 | <0,01 | <0,01 |
| **F2RL2** | 0,0257 | <0,01 | 0,0036 |
| **BDKRB2** | 0,164 | 0,0129 | 0,0012 |
