# Supplementary material for: Identification of a miRNA multi-targeting therapeutic strategy in glioblastoma
Source: Cell Death Dis. 2023 Sep 25;14(9):630. doi: 10.1038/s41419-023-06117-z (PMC10519979; doi:10.1038/s41419-023-06117-z)
Supplement: Supplementary file 12 — Table S5 [file 41419_2023_6117_MOESM12_ESM.docx]

Supplementary Table S5

|  | Hippo pathway | Wnt pathway | MAPK | Autophagy/mTOR | Apoptosis |
| --- | --- | --- | --- | --- | --- |
| miR-17-3p | MOB1B, YAP1, RASSF2, RASSF3, ROCK1 |  | nRAS, DIRAS1, MAPK14 (p38a), BCL2L11 (BIM), MAP4K2, MAPKBP1, | ULK1, AKT3 | caspase 8, caspase 2, caspase 3, BCL2L11 |
| miR-340 | Smad2, ROCK1, FRMD6 | TCF21, FZD3, NFAT5, ROR1 | MAPK14 (p38a), MAPk6 (ERK3), MAPK8IP2 (JIP2), NR2C2(TAK1), MAP3K2 | TOR1B | Capn10, Capn7 (calpain) EIF2S1, |
| miR-222 | Lats2, MOB1B, MOB1A, TEAD1, VGLL4, Samd5, Smad3, Smad2, RUNX1, ROCK1, RASSF4, RASSF3, RASSF2, AMOT, AJUBA,TAOK1, FRMD4B, CD44, Fat4 | Wnt5A, Wnt9A, TCF12, TCF21, APC, FZD3, FZD7, FZD4, FZD1, FZD8, HDAC8, ATF2, RORA | NRAS, MCL1, KRAS, SOS2, RAF1, MAPK6 (ERK3), MAPK8 (JNK1), MAPK1 (ERK2), MAPK14(p38a), MAP3K4, MAP3K5 (ASK1), MAP3K7, MAP3K9, MAP2K2 (MEK2), MAP2K6, MAP3K2, MAP4K4, RASA1, RASAL1, RASAL2, MAPK8IP2, MAPK8IP1, MAPK8IP3, NR2C2, | NRAS, AKT3, TSC1, ATG14, BECN1, PTEN, IRS1, KRAS, PDK1, CDK6, TORB1 | Bcl2L13, caspase2, caspase3, caspase1, caspase9, TRAF1, TRAF5, DDIT3 (CHOP), MAP3K5 (ASK1), TNFRSF(19, 10B, 10A), PARP1, Capn7, EIF2S1 |
