# Supplementary material for: Identification of a miRNA multi-targeting therapeutic strategy in glioblastoma
Source: Cell Death Dis. 2023 Sep 25;14(9):630. doi: 10.1038/s41419-023-06117-z (PMC10519979; doi:10.1038/s41419-023-06117-z)
Supplement: Supplementary file 13 — Supplementary Legends. [file 41419_2023_6117_MOESM13_ESM.docx]

# Supplementary Figure & Table legends

**Supplementary Fig 1**. Kaplan-Meier analysis of the TCGA dataset for miR-17-3p, -340 and 551b expression. (A). Without treatment: left panel, Kaplan-Meier analysis of TCGA dataset for miR-17-3p expression (median=8.494, mean=8.179; SD=1.279, n = 10 miR-17-3plow, n = 12 miR-17-3phigh), middle panel, Kaplan-Meier analysis of TCGA dataset for miR-340 expression (median=7.140, mean=6.851; SD=0.812, n = 12 miR-340low, n = 14 miR-340high), right panel, Kaplan-Meier analysis of TCGA dataset for miR-551b expression (median=7.881, mean=7.346; SD=0.903, n = 17 miR-551blow, n = 21 miR-551bhigh). (B). With treatment: left panel, Kaplan-Meier analysis of TCGA dataset for miR-17-3p expression (median=8.330, mean=7.920; SD=0.980, n = 42 miR-17-3plow, n = 43 miR-17-3phigh), middle panel, KaplanMeier analysis of TCGA dataset for miR-340 expression (median=6.944, mean=6.674;

SD=0.623, n = 40 miR-340low, n = 46 miR-340high), right panel, Kaplan-Meier analysis of

TCGA dataset for miR-551b expression (median=6.711, mean=7.311; SD=1.084, n = 50 miR551blow, n = 48 miR-551bhigh). Kaplan-Meier analysis of TCGA dataset for miR-222 expression, in patients without treatment (C) Kaplan-Meier analysis of TCGA dataset for miR222 expression (median=8.412, mean=9.582; SD=1.996, n = 19 miR-222low, n = 17 miR222high) and in patients with treatment (D) (median=9.546, mean=8.437; SD=2.093, n = 50 miR-222low, n = 50 miR-222high). (E). Correlation between the indicated miRNA expression (data extracted from Gliovis). ns = non-significant.

**Supplementary Fig 2**. (A). The nearest neighbor analysis examines the distances between miR17-3p (top panel), -222 (middle panel), and -340 (bottom panel), and the closest miRNAs. (B). The expression (Log2) of the indicated miRNA was extracted from TCGA dataset.

**Supplementary Fig 3**. (A-N). miRNA expression was determined by qPCR in GDC transfected with miRNA combo (A), non-targeting scrambled control (B) or mimics of miR-173p (C), -340 (D), -551b (E), and -222 antagomir (F) (n=5-6) in Ge518; miRNA combo (G), miR-17-3p (H), -340 (I), and -222 antagomir (J) (n=3-4) in Ge904 and miRNA combo (K), miR-17-3p (L), -340 (M), and -222 antagomir (N) (n=3-4) Ge738. Data are represented as mean ± SEM (*p<0.05, **p<0.01 and ***p<0.001), ns= non-significant.

**Supplementary Fig 4.** (A-N). miRNA expression was determined by qPCR in transfected with miRNA combo (A), or mimics of miR-17-3p (B), -340 (C), and -222 antagomir (D) (n=3-4) in

Ge970,2; miRNA combo (E), miR-17-3p (F), -340 (G), and -222 antagomir (H) (n=3-4) in Ge835. (F-I). Cell viability of Ge518, Ge738, Ge904 and Ge970.2 transiently transfected with non-targeting scrambled control or mimics of miR-17-3p (I), -340 (J), 551b (K) and -222 (L) antagomir was evaluated after three or four days using CellTiter-Glo. Histograms represent the fold change of cell survival for the miR-17-3p, -222, -340 and -Combo versus the miR-Ctrl (n=4-5). (M) Histograms represent the fold change of cell apoptosis and necrosis in Ge518 post-transfection evaluated by the RealTime-Glo Annexin V Apoptosis and Necrosis Assay, (N) Cell-cycle analysis showing the percentage of cells in G0/G1, S, and G2/M in Ge518 cells post-transfection. Data are represented as mean ± SEM (*p<0.05, **p<0.01 and ***p<0.001), ns= non-significant.

**Supplementary Fig 5**. (A-H). Clonogenicity and transmigration of Ge518, Ge738, Ge904 and Ge835 transiently transfected with non-targeting scrambled control or mimics of miR-17-3p (A), -340 (B), -551b (C) and -222 (D) antagomir was determined using the clonogenic assay and, miR-17-3p (E), -340 (F), -551b (G) and -222 (H) antagomir using transwell, respectively. For clonogenicity, histograms represent the fold change of clones formed in each condition versus the miR-Ctrl condition. Representative pictures of 3-4 independent experiments. Scale bar = 1µm. For transmigration, histograms represent the fold change of transmigrated cells through the transwell for each condition versus the miR-Ctrl. Representative pictures of the 3 independent experiments. Scale bar = 1µm. Data are represented as mean ± SEM (*p<0.05, **p<0.01 and ***p<0.001), ns= non-significant.

**Supplementary Fig 6**. The modulation of miR-340, -17-3p and -222 regulates genes involved in several signaling pathways. (A). Functional annotation clustering of gene set enrichment analysis comparing Ge518 transfected with a non-targeting scrambled control or mimics of miR-17-3p, -340, and -222 antagomir. Histograms show the enrichment index of each family of genes. (B-D). Hierarchical clustering of Ge518 transiently transfected with non-targeting scrambled control or mimics of miR-340 (B), -17-3p (C), and -222 antagomir (D) for 24h based on the differentially expressed genes. (E). mRNA was determined by qPCR in Ge518 transfected with a non-targeting scrambled control or mimics of miR-17-3p, -340, and -222 antagomir (n=3). (F). protein was determined by western blot in Ge518 transfected with a non-targeting scrambled control or mimics of miR-17-3p, -340, and -222 antagomir (n=5). Data are represented as mean ± SEM (*p<0.05, **p<0.01 and ***p<0.001), ns= non-significant.

**Supplementary Fig 7**. (A). Venn diagram of comparisons between miRNA potential target genes identified by RNASeq or with TargetScan. Ge518 and Ge970.2 transfected with a nontargeting scrambled control or the combinatorial modulation of miR-340, -17-3p and -222. (B). Functional annotation clustering of gene set enrichment analysis comparing the differentially expressed genes identified in the RNASeq of miR-Combo versus miR-Ctrl and the RNASeq mimics of miR-17-3p, -340, and -222 antagomir. Histograms show the enrichment index of each family of genes. (C). Ge904 PDCs were co-transfected with 3’UTR reporter constructs and either miR-17-3p or miR-340 and luciferase activity was normalized to the respective control.

**Supplementary Table 1**. List of primers used for qRT-PCR.

**Supplementary Table 2.** The identified miRNA listed according to their positive or negative correlation with patient survival.

**Supplementary Table 3.** miRNA expression evaluated by qPCR in all GBM cell lines. Data are represented as mean of 3 independent experiments. Grey rows represent no to low expression; + = fold change <1 normalized on the housekeeping genes; ++ = fold change <2 normalized on the housekeeping genes; +++ = fold change <3 normalized on the housekeeping genes. Mes = mesenchymal; Clas = classical; ProN = proneural; UD = undeterminded.

**Supplementary Table 4.** P Values of the Kaplan-Meier analysis of the TCGA, Rembrant, and Gravendeel datasets for the listed genes.

**Supplementary Table 5.** List of the potential target genes and the family of genes for miR-173p, -340, and -222 identified via TargetScan.
