# Supplementary material for: Identification of a miRNA multi-targeting therapeutic strategy in glioblastoma
Source: Cell Death Dis. 2023 Sep 25;14(9):630. doi: 10.1038/s41419-023-06117-z (PMC10519979; doi:10.1038/s41419-023-06117-z)

Figure 2

- GDC 518 – PARP tot / C-PARP :

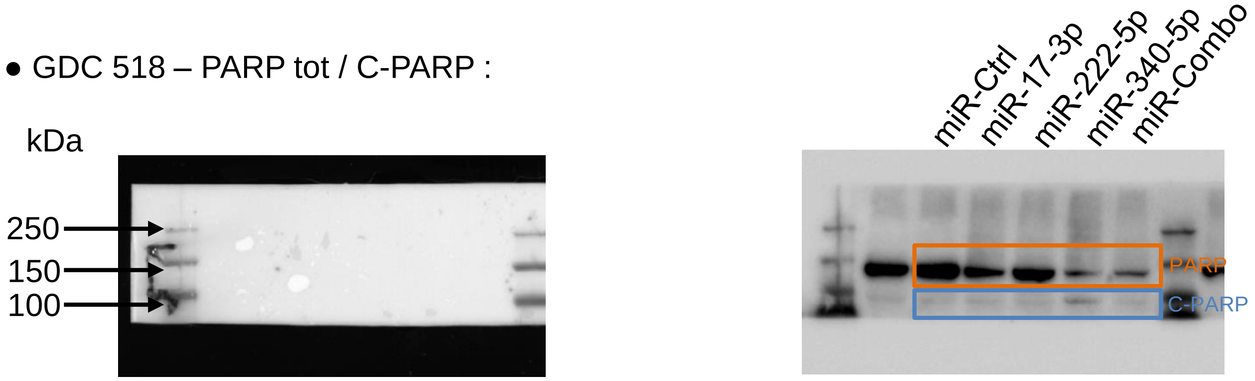

- GDC 518 – C-Cas3:

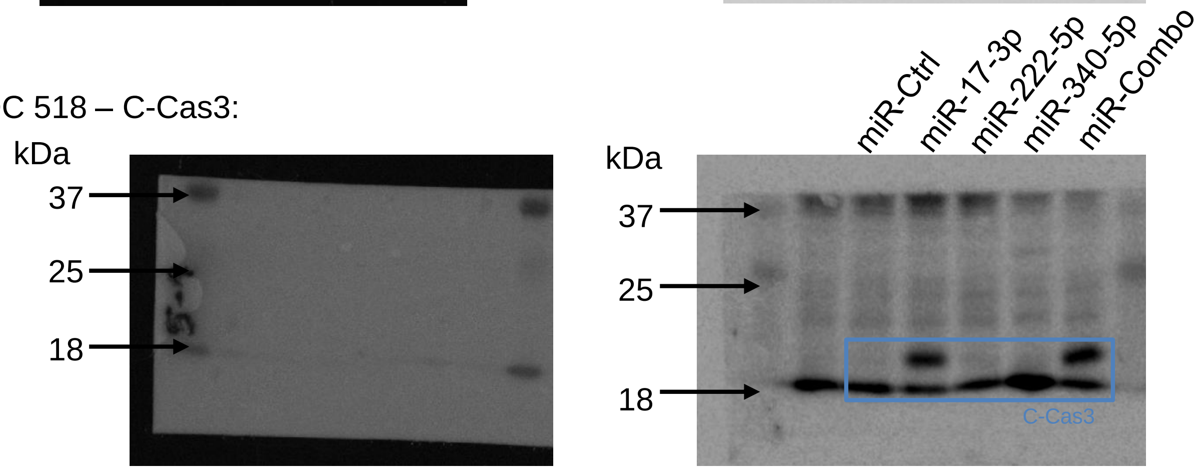

- GDC 518 – Cas3 tot:

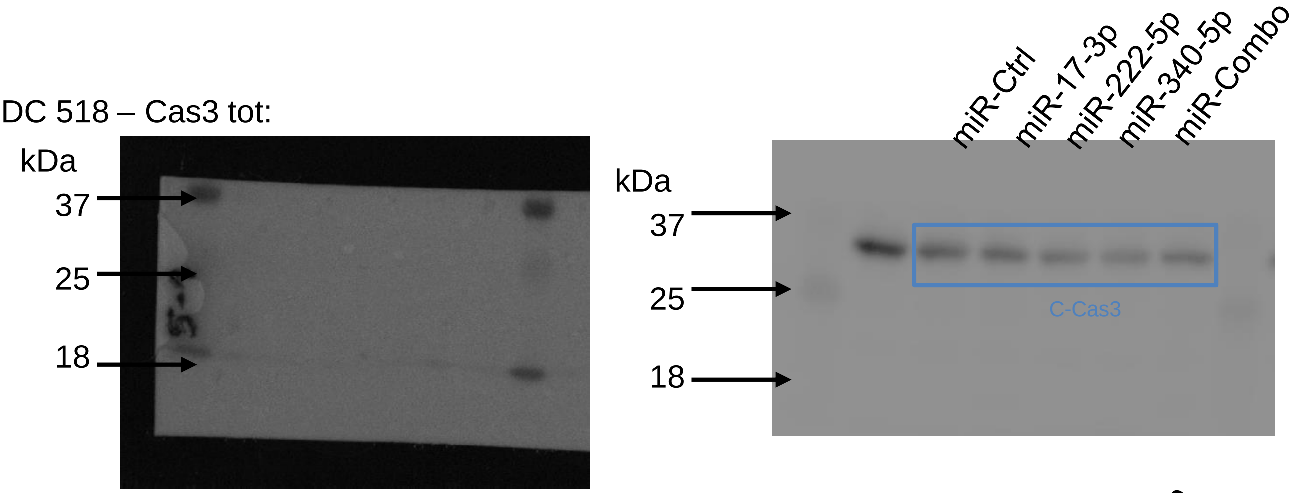

- GDC 835 – Cas3 tot:

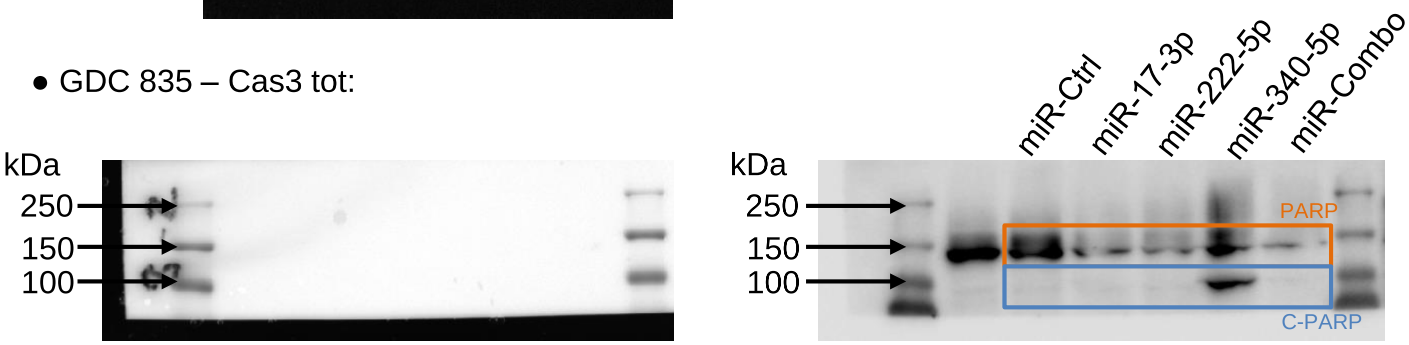

Figure 2

• GDC 835 – C-Cas3:

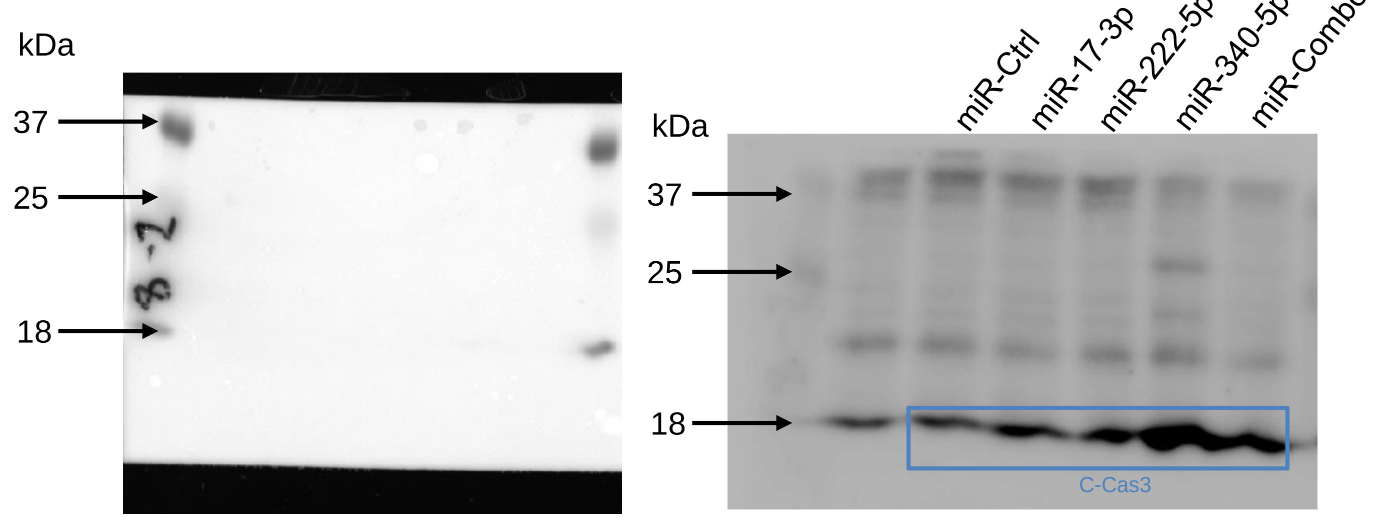

• GDC 835 – Cas3 tot:

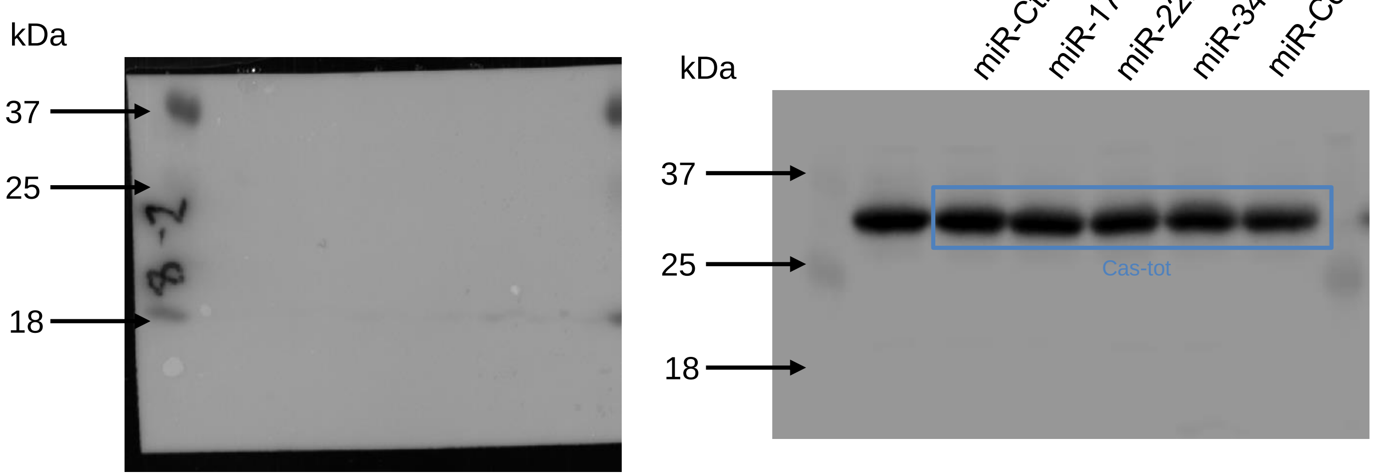

• GDC 904 – PARP tot / C-PARP :

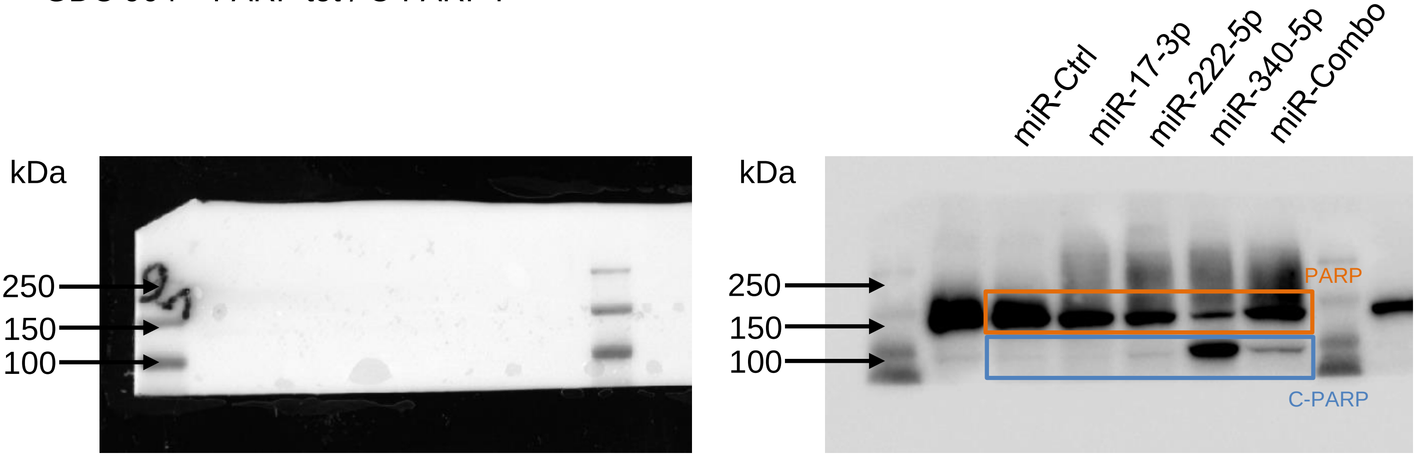

Figure 2

• GDC 904 – C-Cas3:

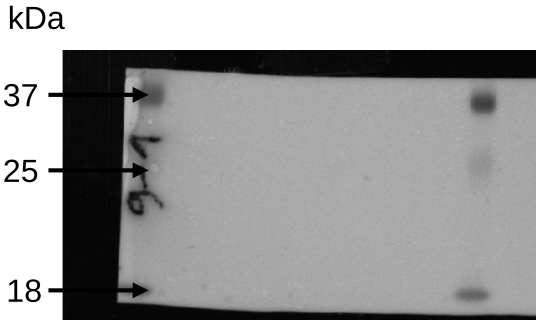

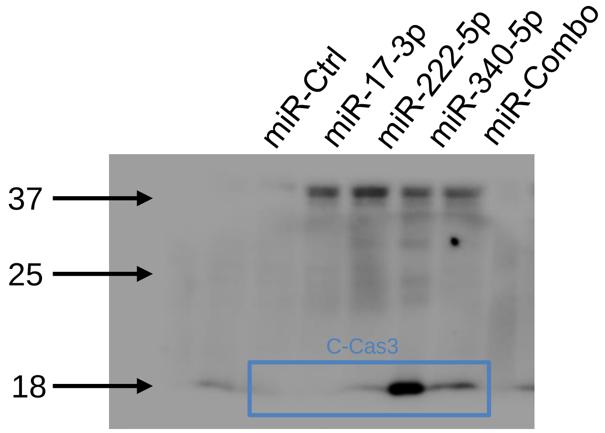

• GDC 904 – Cas3 tot:

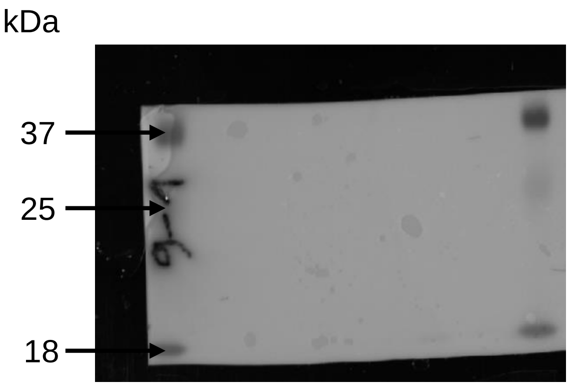

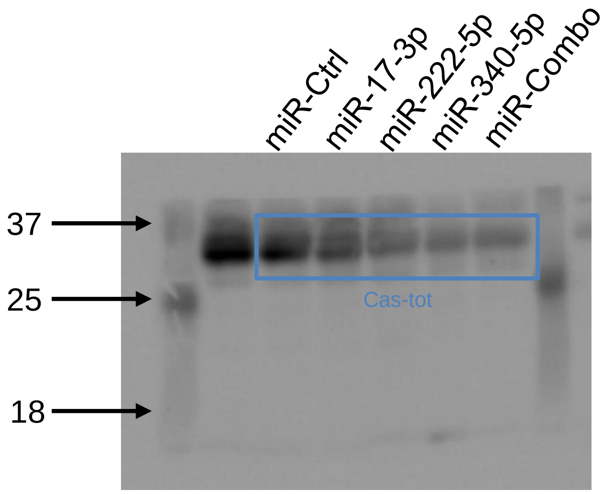

• Actin :

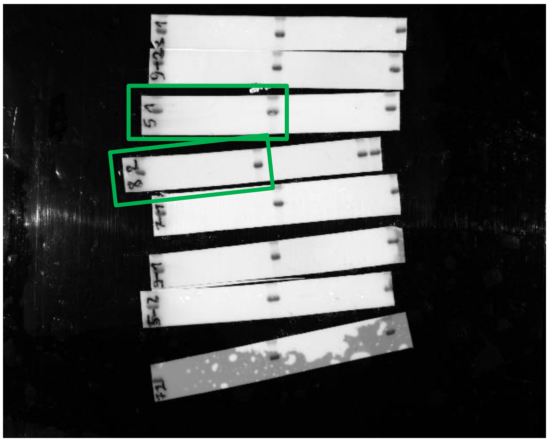

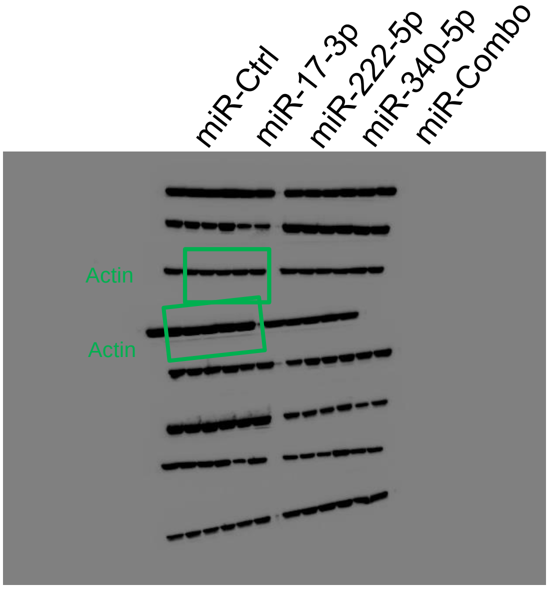

Figure 4

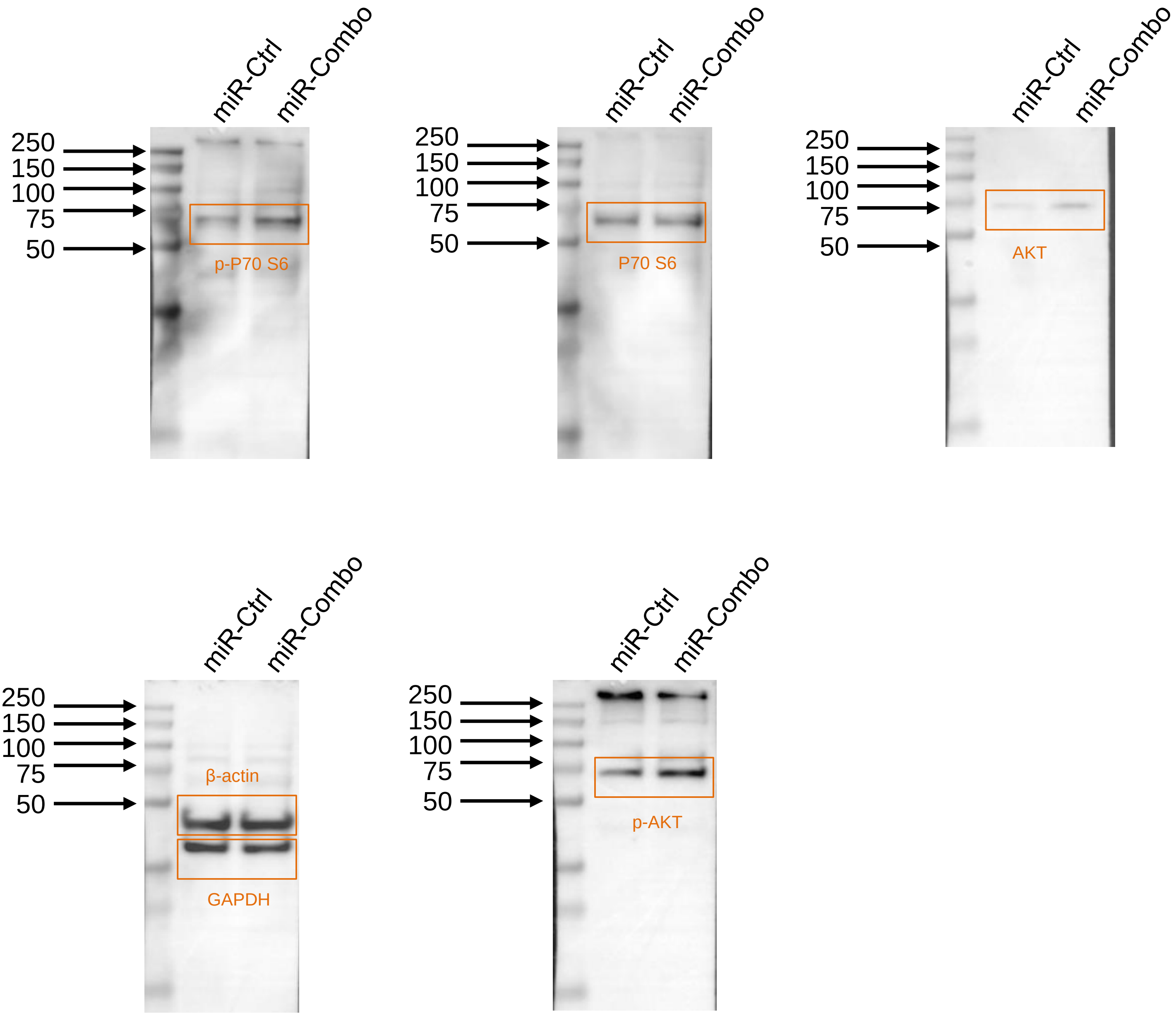
miR-Ctrl
miR-Combo
250
150
100
75
50
p-P70 S6
P70 S6
AKT
β-actin
GAPDH
p-AKT

Figure 6

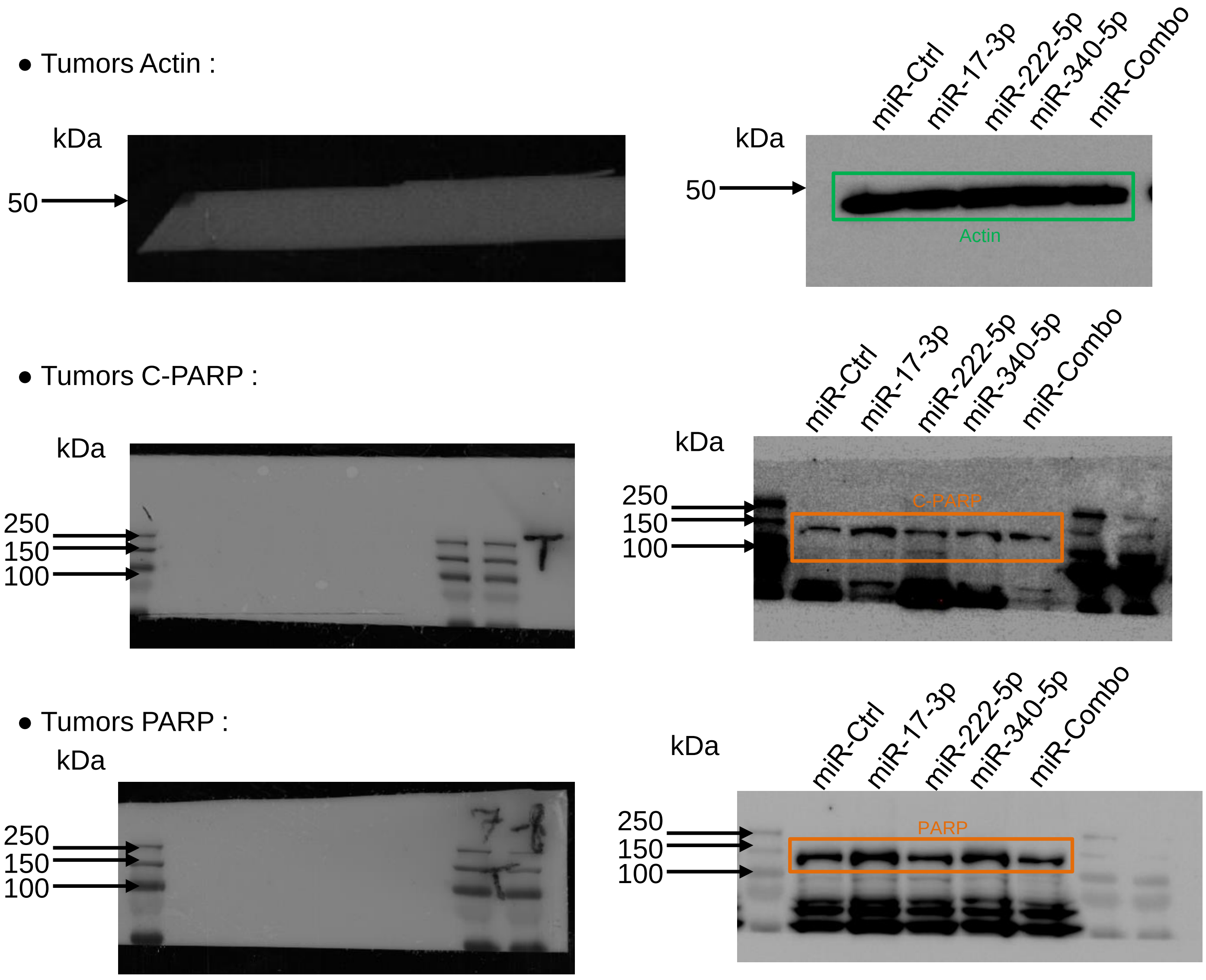
• Tumors Actin :
kDa
50
miR-Ctrl
miR-17-3p
miR-222-5p
miR-340-5p
miR-Combo
kDa
50
Actin
• Tumors C-PARP :
kDa
250
150
100
miR-Ctrl
miR-17-3p
miR-222-5p
miR-340-5p
miR-Combo
kDa
250
150
100
C-PARP
• Tumors PARP :
kDa
250
150
100
miR-Ctrl
miR-17-3p
miR-222-5p
miR-340-5p
miR-Combo
kDa
250
150
100
PARP

Figure 6

• Tumors cas3-C :

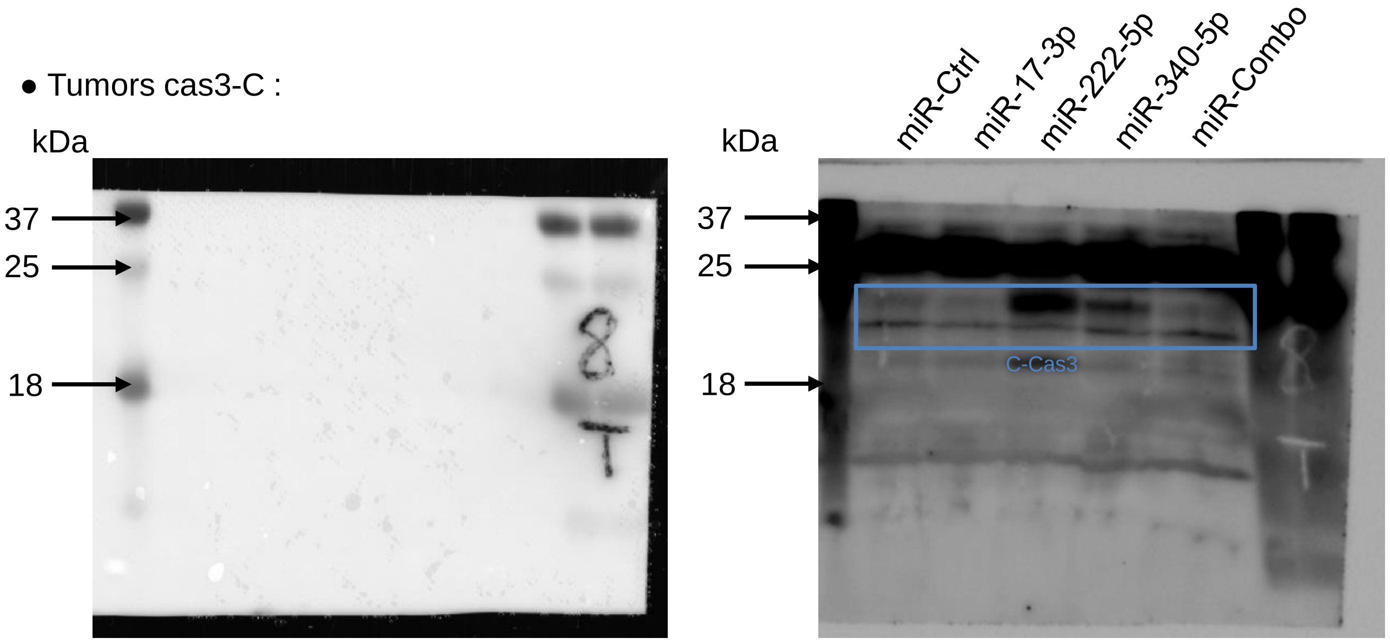

• Tumors cas3-tot :

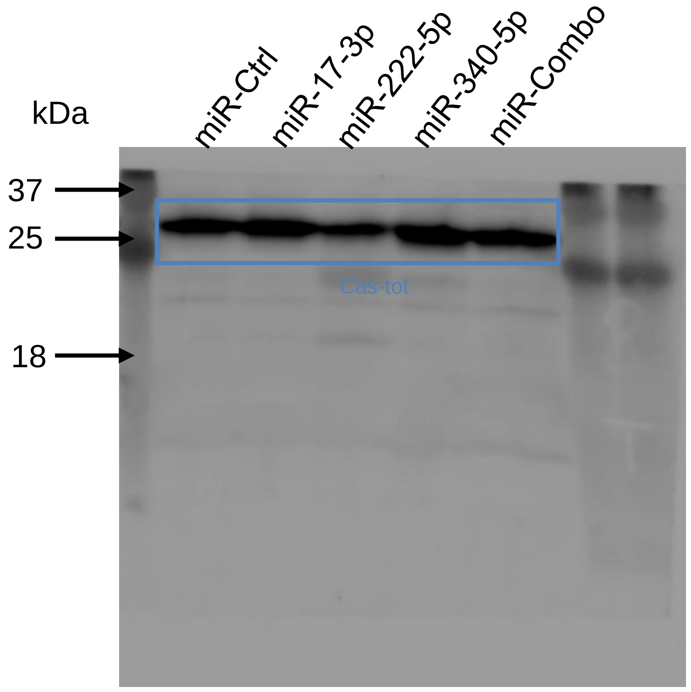

Supplementary Figure 4

- GDC 970.2 C-cas3 :

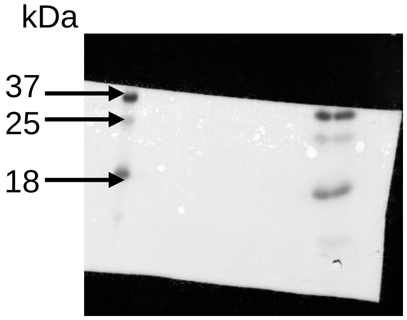

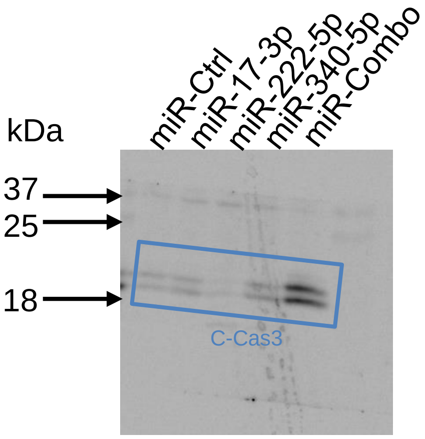

- GDC 970.2 C-cas3 :

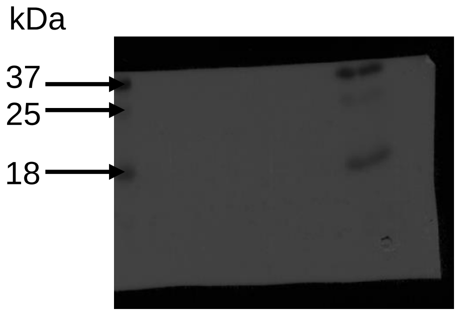

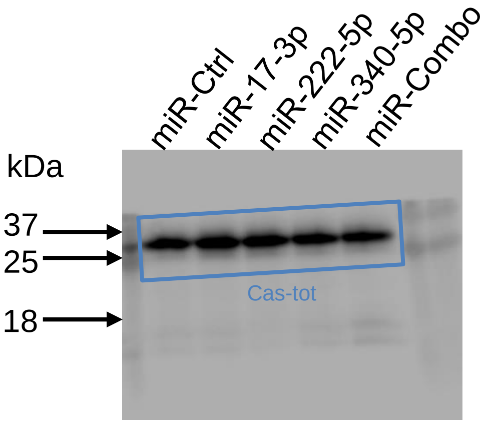

- GDC 970.2 Actin :

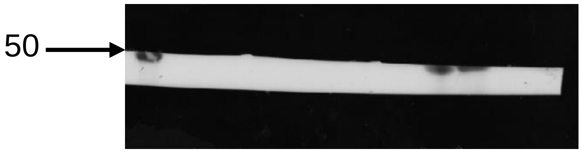

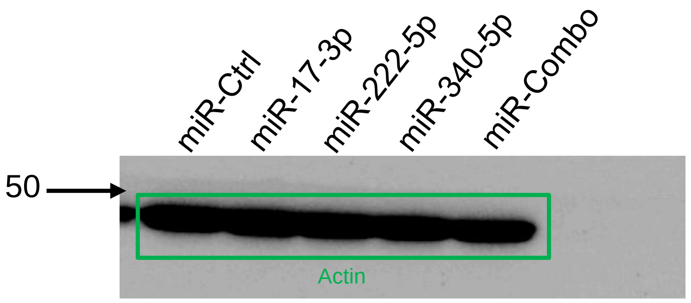

- GDC 738 C-cas3 :

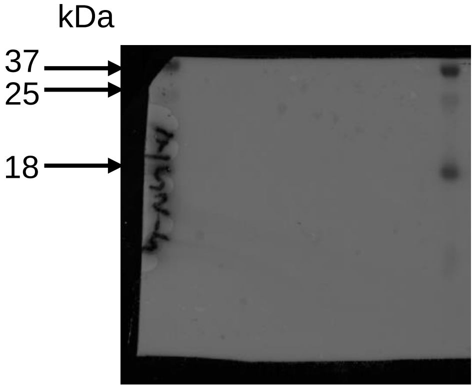

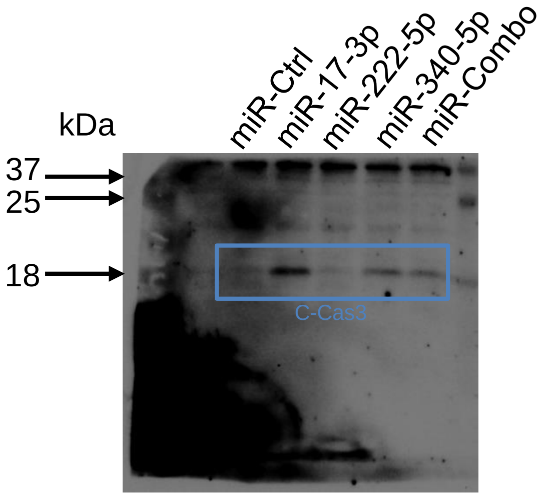

- GDC 738 tot-cas3 :

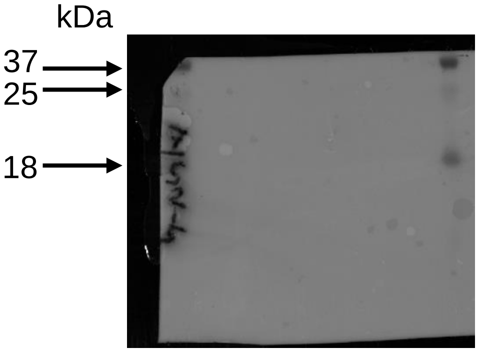

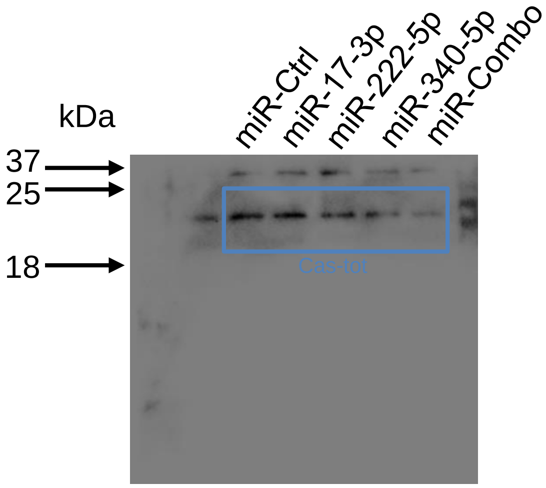

- GDC 738 Actin :

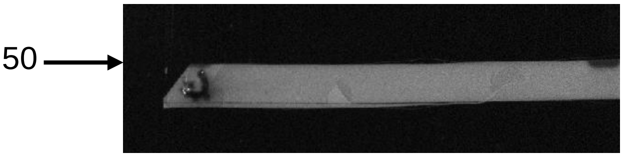

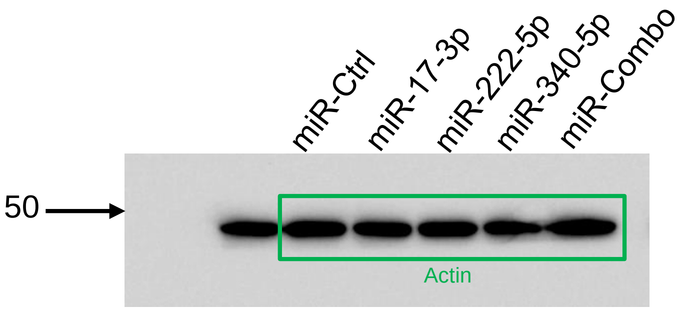

- Tumors Vimentin :

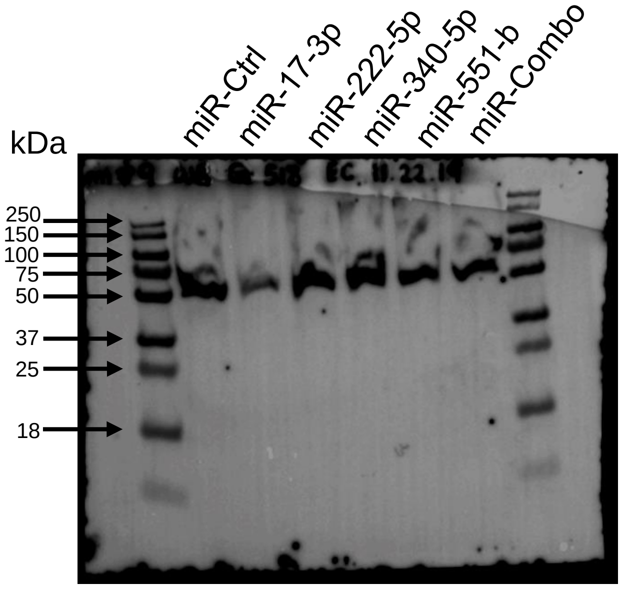

- Tumors Actin :

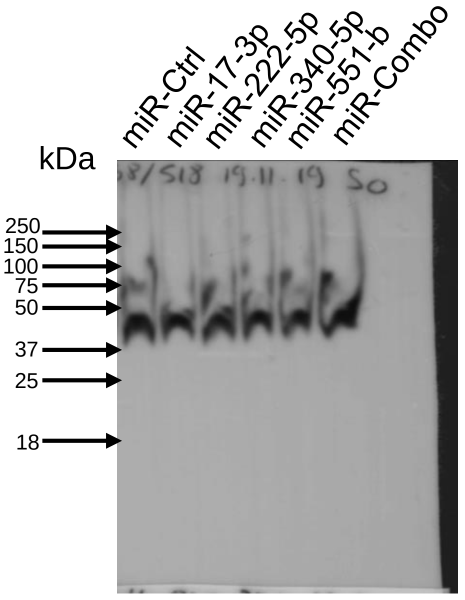

- Tumors Litaf / Actin :

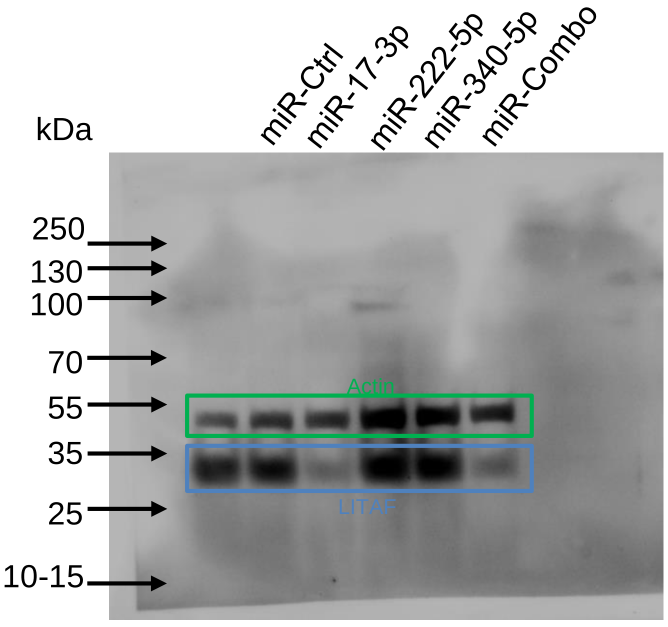

Supplement: Supplementary file 14 — Original Data File [file 41419_2023_6117_MOESM14_ESM.pdf]
